# DNA Replication Vulnerabilities Render Ovarian Cancer Cells Sensitive to Poly(ADP-Ribose) Glycohydrolase Inhibitors

**DOI:** 10.1016/j.ccell.2019.02.004

**Published:** 2019-03-18

**Authors:** Nisha Pillay, Anthony Tighe, Louisa Nelson, Samantha Littler, Camilla Coulson-Gilmer, Nourdine Bah, Anya Golder, Bjorn Bakker, Diana C.J. Spierings, Dominic I. James, Kate M. Smith, Allan M. Jordan, Robert D. Morgan, Donald J. Ogilvie, Floris Foijer, Dean A. Jackson, Stephen S. Taylor

**Affiliations:** 1 Division of Cancer Sciences, Faculty of Biology, Medicine and Health, University of Manchester, Manchester Cancer Research Centre, 555 Wilmslow Road, Manchester M20 4GJ, UK; 2 European Research Institute for the Biology of Ageing (ERIBA), University of Groningen, University Medical Center Groningen, 9713 AV Groningen, the Netherlands; 3 Drug Discovery Unit, Cancer Research UK Manchester Institute, University of Manchester, Wilmslow Road, Manchester, M20 4BX, UK; 4 The Christie NHS Foundation Trust, Wilmslow Road, Manchester M20 4BX, UK; 5 Division of Molecular and Cellular Function, Faculty of Biology, Medicine and Health, University of Manchester, Michael Smith Building, Oxford Road, Manchester M13 9PL, UK

**Keywords:** DNA damage, PARG, PARP, replication catastrophe, γH2AX, TIMELESS, HGSOC

## Abstract

Inhibitors of poly(ADP-ribose) polymerase (PARP) have demonstrated efficacy in women with *BRCA*-mutant ovarian cancer. However, only 15%–20% of ovarian cancers harbor *BRCA* mutations, therefore additional therapies are required. Here, we show that a subset of ovarian cancer cell lines and *ex vivo* models derived from patient biopsies are sensitive to a poly(ADP-ribose) glycohydrolase (PARG) inhibitor. Sensitivity is due to underlying DNA replication vulnerabilities that cause persistent fork stalling and replication catastrophe. PARG inhibition is synthetic lethal with inhibition of DNA replication factors, allowing additional models to be sensitized by CHK1 inhibitors. Because PARG and PARP inhibitor sensitivity are mutually exclusive, our observations demonstrate that PARG inhibitors have therapeutic potential to complement PARP inhibitor strategies in the treatment of ovarian cancer.

## Highlights

• Ovarian cancer cells show differential sensitivity to PARP and PARG inhibitors
• PARG inhibitor sensitivity is due to an underlying DNA replication vulnerability
• PARG inhibition blocks fork restart leading to persistent replication stress
• PARG inhibition sensitizes cells to drugs targeting the CHK1 kinase

## Graphical Abstract

**Figure:**
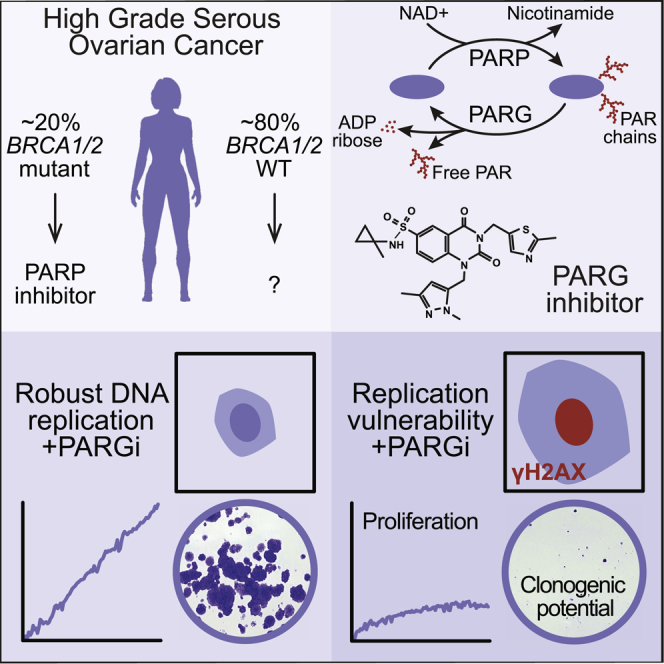

## Teaser

Pillay et al. show that ovarian cancer cells respond to inhibition of poly(ADP-ribose) (PAR) polymerase and inhibition of PAR glycohydrolase (PARG) differently; sensitivity to the latter is due to persistent fork stalling and replication catastrophe. Inhibiting CHK1 sensitizes tumor cells to PARG inhibition.

## Significance

**PARP inhibitors are efficacious treatments for *BRCA*-mutant high-grade serous ovarian cancer. However, most ovarian cancers do not have *BRCA* mutations and are unlikely to respond to PARP inhibitors, therefore additional therapeutic strategies are required. We show that a subset of preclinical ovarian cancer models is sensitive to pharmacological inhibition of PARG, the glycohydrolase that counterbalances PARP activity. Sensitivity arises due to an underlying DNA replication vulnerability such that upon PARG inhibition, stalled DNA replication forks fail to restart, leading to replication catastrophe. Inhibiting PARG also sensitizes cells to drugs targeting the DNA damage response checkpoint kinase CHK1. Because PARP and PARG inhibitor sensitivity does not overlap, PARG inhibitors could offer an additional treatment strategy for ovarian cancer.**

## Introduction

Personalized medicine offers great promise for improving the efficacy of cancer treatment strategies. Indeed, therapeutic agents inhibiting oncogenic drivers such as BRAF, EGFR, and HER2 have allowed systemic anticancer therapy to target tumors directly, with considerable success (La Thangue and Kerr, 2011). Unfortunately, this paradigm is challenging in high-grade serous ovarian cancer (HGSOC) where there is a paucity of actionable driver mutations (The Cancer Genome Atlas Research Network, 2011, Patch et al., 2015). However, the high frequency of DNA damage repair (DDR) defects opens up an alternative strategy, namely synthetic lethality, pioneered by the use of inhibitors targeting poly(ADP-ribose) polymerase (PARP) 1 and 2 (Bryant et al., 2005, Farmer et al., 2005). Indeed, PARP inhibitors have shown impressive efficacy in women with HGSOC, as both maintenance treatment following platinum chemotherapy and as single agents (Mirza et al., 2016, Coleman et al., 2017, Pujade-Lauraine et al., 2017). Thus, there has been a rapid escalation of PARP inhibitors in clinical use, with three agents currently licensed, namely olaparib, niraparib, rucaparib (Ashworth and Lord, 2018).

The PARP family comprises 17 members, which control a wide array of cellular processes, with PARP1/2 intimately involved in DDR (Gibson and Kraus, 2017). Following single-strand breaks, these enzymes mobilize to sites of damage and catalyze the assembly of branched poly(ADP-ribose) (PAR) chains on acceptor proteins, thereby facilitating recruitment of repair factors (Rouleau et al., 2010, Helleday, 2011, Ray Chaudhuri and Nussenzweig, 2017). When PARP1/2 are inhibited, cells become dependent on parallel pathways to maintain genome integrity, in particular homologous recombination (HR). When HR is compromised, for example, due to mutations in *BRCA1* or *BRCA2*, cells are rendered exquisitely sensitive to PARP1/2 inhibition (Bryant et al., 2005, Farmer et al., 2005), a mechanism dependent in part on drug-mediated PARP trapping (Murai et al., 2012, Hopkins et al., 2015). Indeed, the presence of a *BRCA* mutation is a clinically validated predictive biomarker of PARP inhibitor sensitivity (Moore et al., 2018), and this has led to widespread implementation of germline and tumor *BRCA* testing to identify patients likely to benefit from PARP inhibitors. However, as only 15%–20% of HGSOC possess a *BRCA* mutation (The Cancer Genome Atlas Research Network, 2011, Patch et al., 2015), there is a pressing need to develop additional therapeutic strategies.

In response to DNA damage and activation of PARP1/2, the subsequent degradation of the PAR chains is required for repair processes to be completed (Gibson and Kraus, 2017). This catabolic step is performed by poly(ADP-ribose) glycohydrolase (PARG), a macrodomain protein with exo- and endo-glycohydrolase activity that liberates free ADP-ribose and PAR chains, respectively (Rack et al., 2016). Consequently, the balance between PARP and PARG activity is essential for efficient DDR (Barkauskaite et al., 2013, Gogola et al., 2018). Note, however, that PARG's role is not restricted to the DDR; indeed PARG influences multiple cellular functions including chromatin modulation, transcription, DNA replication, mitochondrial function, and apoptosis (Feng and Koh, 2013, Gibson et al., 2016, Rack et al., 2016).

In light of PARP1/2 being clinically validated targets and PARG also being intimately involved in DDR, and because the enzyme's catalytic pocket is amenable to inhibition with small molecules (Dunstan et al., 2012), PARG represents an attractive synthetic lethality target. To test this hypothesis, we developed the PARG inhibitor, PDD00017273, a quinazolinedione that inhibits PARG with an *in vitro* half maximal inhibitory concentration of 26 nM and stabilizes cellular PAR chains with an half maximal effective concentration of 37 nM (James et al., 2016). Importantly, PDD00017273 is devoid of activity against PARP1 and the ARH3 glycohydrolase. Of several breast cancer lines tested, most were insensitive to PDD00017273, including those with *BRCA* mutations, while a *BRCA*-proficient line was particularly sensitive. While this suggests PARG inhibitors may be differentiated from PARP inhibitors, the mechanism responsible for PARG inhibitor sensitivity and the wider impact of these initial observations remains to be determined.

To evaluate the potential of PARG inhibitors in the context of HGSOC, we set out to ask a number of specific questions. Are preclinical HGSOC models sensitive to PARG inhibition? If so, what is the underlying mechanism, and can this insight inform the design of predictive biomarkers and rational combination strategies? And finally, does PARG inhibition show efficacy in ovarian cancer cells resistant to PARP inhibitors?

## Results

### Identification of Ovarian Cancer Cell Lines with Differential PARG Inhibitor Sensitivity

**Figure 1:**
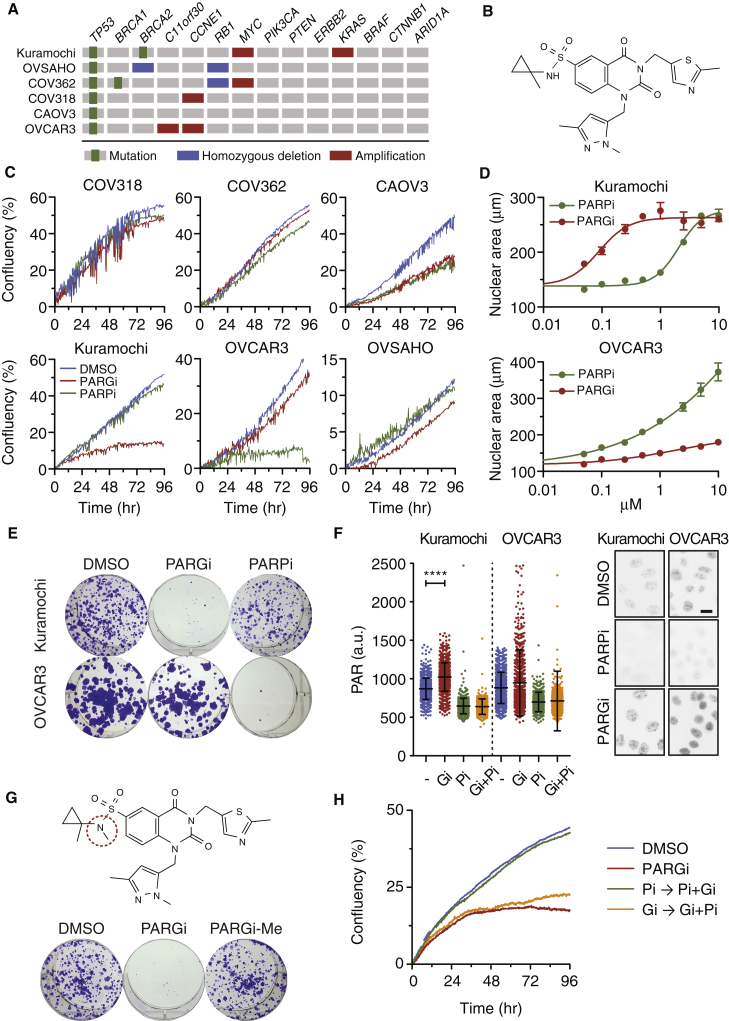
Ovarian Cancer Cells Display Differential Sensitivity to PARG and PARP Inhibitors (A) Mutation profiles of selected ovarian cancer cell lines (Domcke et al., 2013). (B) Chemical structure of PDD00017273. (C) Proliferation of ovarian cancers cells pre-treated for 48 h with 1 μM PARGi or 1 μM olaparib (PARPi), then analyzed by time-lapse imaging for a further 96 h in the continued presence of inhibitor. (D) Nuclear area in response to increasing concentrations of inhibitors. Values represent mean ± SEM from two technical replicates. (E) Colony formation in the continuous presence of inhibitors at 1 μM. (F) Immunofluorescence images and quantitation showing PAR levels in the presence of indicated inhibitors at 1 μM. Scale bar, 10 μm. Values derived from 1,000 cells and bars indicate mean ± SD and are representative of two biological experiments ^∗∗∗∗^p < 0.0001. (G) Chemical structure of PDD00031704 (PARGi-Me), an inactive analog of PARGi, and colony formation assay using both PARGi and PARGi-Me at 1 μM. (H) Proliferation curves of Kuramochi cells pre-treated with 1 μM PARPi for 48 h then exposed to 1 μM PARGi in the continued presence of 1 μM PARPi (Pi→Pi + Gi), or pre-treated with 1 μM PARGi for 48 h then exposed to 1 μM PARPi in the continued presence of 1 μM PARGi (Gi→Gi + Pi), or exposed to 1 μM PARGi alone continuously, then analyzed by time-lapse imaging for a further 96 h. Values represent the mean from three technical replicates. See also Figure S1.

To determine whether PARG inhibitors might open up therapeutic opportunities in ovarian cancer, we assembled a panel of six ovarian cancer cell lines with genomic features that reflect HGSOC, namely Kuramochi, OVSAHO, COV362, COV318, CAOV3, and OVCAR3 (Figure S1A). All six lines harbor *TP53* mutations and extensive copy number aberrations (Domcke et al., 2013). In addition, three are reported to have *BRCA1* or *BRCA2* mutations, two have amplified *MYC*, and two have amplified *CCNE1* (Figure 1A). To inhibit PARG, we used the PARG inhibitor PDD00017273 (James et al., 2016), hereafter PARGi (Figure 1B), and compared it with the PARP1/2 inhibitor olaparib (Menear et al., 2008), hereafter PARPi. To assess relative sensitivity, we monitored proliferation in the continuous presence of inhibitors. While COV318, COV362, CAOV3, and OVSAHO proliferated in both inhibitors, Kuramochi and OVCAR3 displayed differential sensitivities; while Kuramochi was suppressed by PARGi, OVCAR3 was suppressed by PARPi (Figure 1C). Consistently, Kuramochi cells appeared morphologically normal in PARPi while in PARGi they adopted a “fried egg” morphology, with round cytoplasms and enlarged nuclei (Figures 1D and S1B). Importantly, this differential sensitivity manifested over a range of drug concentrations (Figure S1C) and in longer term colony formation assays (Figure 1E). PARGi stabilized PAR chains in both Kuramochi and OVCAR3, and this was blocked by co-treatment with the PARPi (Figures 1F and S1D), indicating target engagement in both lines. Two observations indicate that the anti-proliferative effect of PARGi on Kuramochi was due to inhibition of PARG. Firstly, an N-methylated analog of PARGi with minimal activity *in vitro* (James et al., 2016) was inactive in the colony formation assay (Figure 1G). Secondly, pre-exposing Kuramochi to PARPi prevented the PARGi effect (Figure 1H), indicating that the efficacy of PARGi requires assembly of PAR chains. Thus, we conclude that a subset of ovarian cancer cell lines is sensitive to PARGi and shows differential PARPi/PARGi sensitivity.

### PARGi Blocks Mitotic Entry

**Figure 2:**
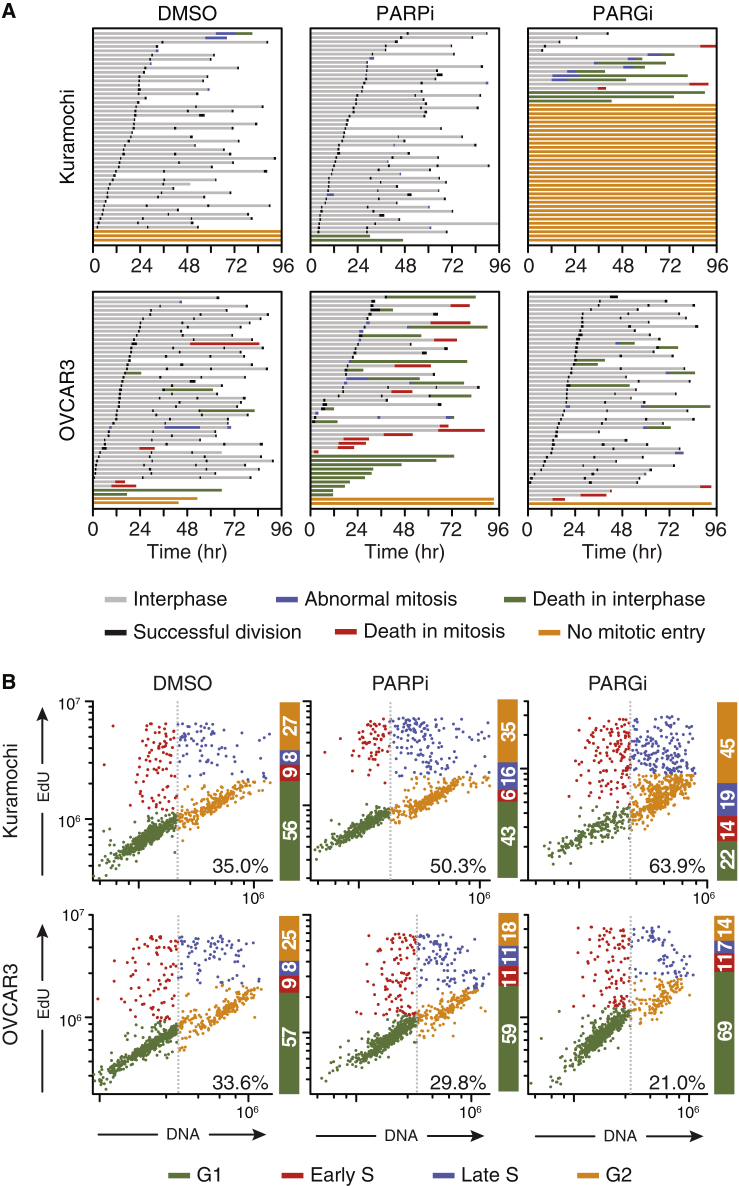
PARG Inhibition Blocks Entry into Mitosis (A) Cell fate profiles of cells exposed to 1 μM PARGi or 1 μM PARPi for 48 h then imaged by time-lapse microscopy in the continued presence of the inhibitors for a further 96 h. T = 0 indicates when imaging started. Each horizontal bar represents a single cell, with the colors indicating cell behavior. At least 50 cells were analyzed per condition. (B) Cell-cycle profiles determined by immunofluorescence imaging of DAPI-stained nuclei following a 1 h EdU pulse to identify S-phase cells. Lower right values indicate percentage of cells in late S phase (blue) and G_2_ (orange). Values derived from 880 cells per condition. See also Figure S2.

To understand the differential sensitivity, we analyzed Kuramochi and OVCAR3 by time-lapse microscopy to determine cell fate upon exposure to PARGi and PARPi. The vast majority of untreated and PARPi-treated Kuramochi cells underwent multiple divisions, whereas most of PARGi-treated cells were blocked in interphase (Figure 2A). By contrast, the majority of untreated and PARGi-treated OVCAR3 cells underwent multiple divisions, whereas cell death increased markedly when treated with PARPi. Thus, while the PARPi effect on OVCAR3 is largely cytotoxic, the PARGi effect on Kuramochi is largely cytostatic. Cell-cycle analysis showed that while PARPi had a modest effect on OVCAR3, PARGi had a substantial effect on Kuramochi, increasing the proportion of cells in late S phase and G_2_ (Figures 2B and S2A). Note that despite the mitotic entry block, PARGi-treated Kuramochi cells progressed through S phase, as demonstrated by 5-ethynyl-2′-deoxyuridine (EdU) incorporation, indicating activation of S and G_2_/M checkpoints rather than a G_1_/S checkpoint. Indeed, inhibiting the WEE1 kinase with AZD1775, hereafter WEE1i, alleviated the PARGi-induced block, driving Kuramochi cells into aberrant mitoses and increasing apoptosis (Figure S2B). Thus, we conclude that PARGi blocks Kuramochi prior to mitotic entry due to activation of S phase and G_2_/M checkpoint controls. In turn, this provides a possible explanation for the “fried egg” morphology; when breast epithelial cells undergo prolonged S-phase arrest, they undergo a reversible senescent-like phenotype with large flattened nuclei (Maya-Mendoza et al., 2014).

### PARG Inhibition Induces Replication Catastrophe

**Figure 3:**
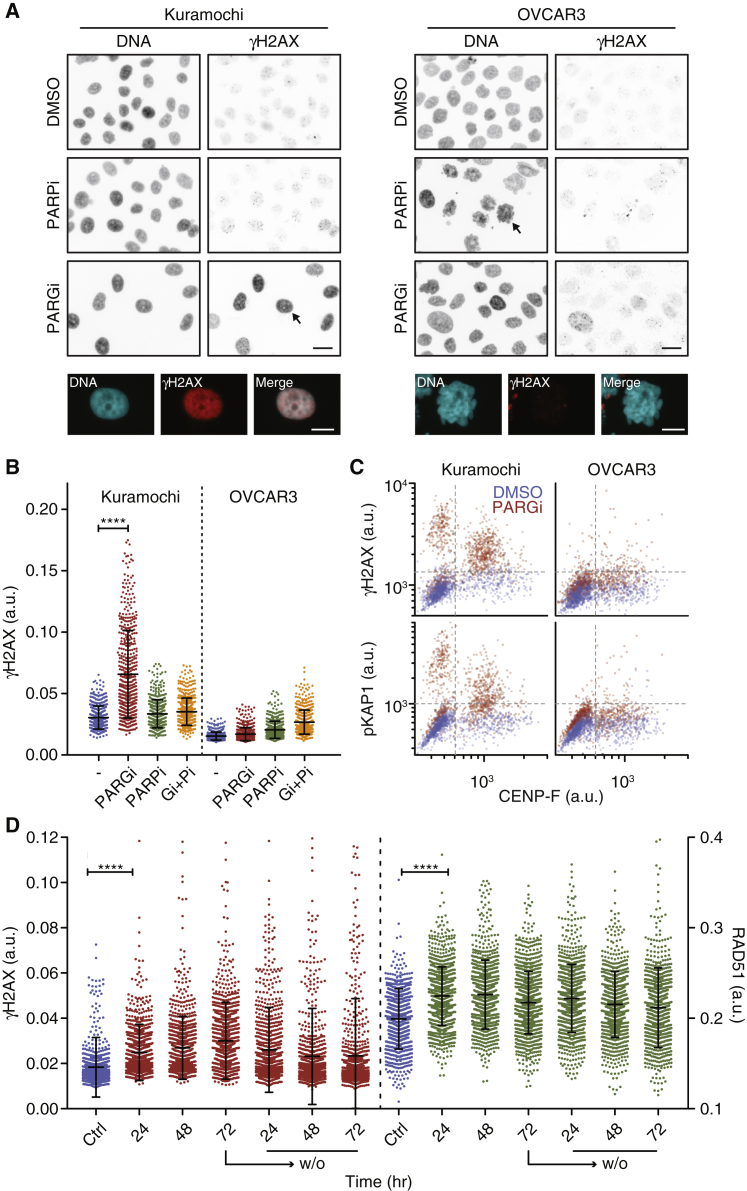
PARG Inhibition Induces Replication Catastrophe (A) Immunofluorescence images of cells treated for 96 h with 1 μM PARGi or 1 μM PARPi then stained to detect γH2AX. Scale bar, 20 μm (top). Arrows indicate the cells shown in enlargements at the bottom. Scale bar, 10 μm (bottom). (B) Quantitation of γH2AX staining in Kuramochi and OVCAR3 cells. Values derived from 500 cells and bars represent the mean ± SD and are representative of two independent experiments. (C) Scatterplot quantitating γH2AX, phospho-KAP1 and CENP-F staining in 1,000 cells per condition. (D) Time course quantitating γH2AX and RAD51 staining in 1,000 Kuramochi cells exposed to 1 μM PARGi for up to 72 h then following washout. Bars show the mean ± SD and represent two independent experiments. ^∗∗∗∗^p < 0.0001. See also Figure S3.

To determine whether DNA damage was responsible for the PARGi-induced cell-cycle block in Kuramochi, we analyzed γH2AX, an indirect biomarker for double-strand breaks and replication stress-induced defects (Burma et al., 2001, Ward and Chen, 2001). While both PARPi and PARGi had minimal effect on γH2AX in OVCAR3, PARGi had a dramatic effect on Kuramochi cells, inducing a chromatin-bound pan-nuclear staining pattern (Figures 3A and S3A). Importantly, this pan-nuclear γH2AX did not manifest upon co-exposure to PARPi (Figure 3B), indicating that it is induced by stabilizing PAR chains, rather than off-target drug effects. Indeed, RNAi-mediated repression of PARG also induced pan-nuclear γH2AX in Kuramochi (Figure S3B). γH2AX foci can indicate double-strand breaks, which activate ATM-dependent repair pathways (Burma et al., 2001). Consistently, PARGi induced phosphorylation of the ATM substrate KAP1 (Figure 3C). However, OVCAR3 and Kuramochi both induced RAD51 foci in response to ionizing radiation (Figure S3C), indicating that differences in HR are unlikely to account for the differential sensitivity. Indeed, the pan-nuclear γH2AX phenotype observed more likely reflects replication catastrophe, a phenomenon whereby prolonged replication stress exhausts levels of the heterotrimeric single-stranded DNA binding complex RPA, in turn leading to genome-wide replication fork collapse (Toledo et al., 2013, Toledo et al., 2017). Indeed, short hairpin RNA (shRNA)-mediated inhibition of PARG in HeLa cells was previously shown to inhibit DNA replication and induce γH2AX (Ray Chaudhuri et al., 2015). Consistent with PARGi inducing replication stress, CHK1 was phosphorylated on serine 345 in PARGi-treated Kuramochi cells (Figure S3D). Moreover, pan-nuclear γH2AX was restricted to cells in S phase and G_2_ (Figure S3D) and was suppressed when cells were blocked in G_1_ (Figure S3E). To study the dynamics of the γH2AX response, Kuramochi cells were analyzed for 72 h before and after PARGi washout. When exposed to PARGi, γH2AX-positive cells increased then decreased following washout, approaching basal numbers by the end of the experiment (Figure 3D). Interestingly, while RAD51-positive cells also increased upon PARGi exposure, the decrease following washout was less pronounced, suggesting persistent DNA damage despite the decline of γH2AX. Consistently, while a 24-h pulse of PARGi had little effect on viability, a 72-h pulse was severely detrimental (Figure S3F). A possible explanation for these observations is that PARGi induces replication stress, and while cells can recover from brief periods without PARG activity, prolonged PARGi exposure leads to pervasive replication fork collapse, irreparable DNA damage, and diminished clonogenic potential.

### DNA Replication Factors Are Synthetic Lethal with PARG Inhibition

**Figure 4:**
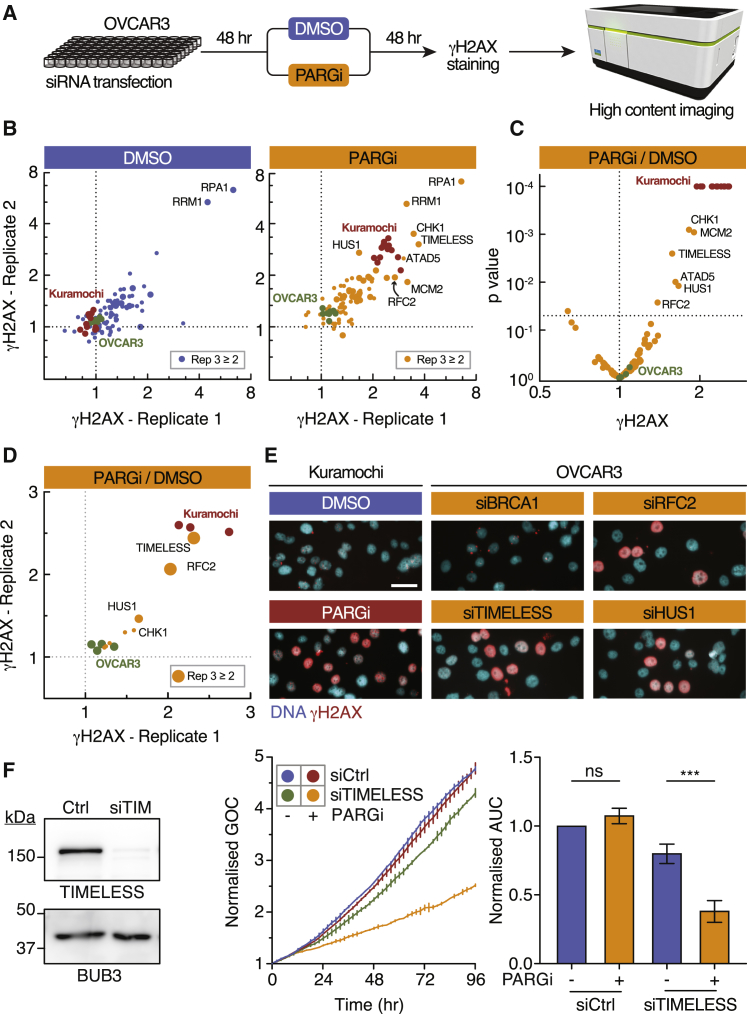
DNA Replication Factors Are Synthetic Lethal with PARG Inhibition (A) Workflow of siRNA library screen. (B) Primary screen plotting γH2AX for replicates 1 and 2 with values showing fold change relative to a non-targeting siRNA control. Values ≥2 in replicate 3 are denoted by a larger symbol. (C) Volcano plot showing γH2AX fold change and p value; values derived from three independent experiments. (D) Secondary screen with independent siRNAs plotting γH2AX fold change for replicates 1 and 2. (E) Immunofluorescence images of siRNA-transfected OVCAR3 cells exposed to 1 μM PARGi and stained to detect γH2AX. Scale bar, 50 μm. (F) Immunoblot, nuclear proliferation (green object count) curves, and quantification of area under curve (AUC) of OVCAR3 cells following siTIMELESS and exposure to 1 μM PARGi. BUB3 is used as a loading control. Bar graph shows the mean ± SEM derived from three independent experiments. ^∗∗∗^p < 0.001. See also Figure S4.

To understand why Kuramochi are PARGi sensitive, we performed a small interfering RNA (siRNA) library screen to identify PARGi synthetic lethal targets in PARGi-resistant OVCAR3 cells, using elevated pan-nuclear γH2AX as an endpoint biomarker (Figure 4A). The primary screen focused on 74 genes previously shown to be synthetic lethal with olaparib in *BRCA1* and *BRCA2*-proficient MCF7 breast cancer cells (Bajrami et al., 2014). In the absence of inhibitor, Kuramochi, OVCAR3, and the majority of siRNA-transfected OVCAR3 cells had low γH2AX (Figure 4B). Two exceptions stood out; siRNAs targeting RPA1 and RRM1, which are components of RPA and ribonucleotide reductase (RNR), respectively, were sufficient to induce strong pan-nuclear γH2AX, consistent with this phenomenon reflecting replication catastrophe. Note that inhibition of RNR causes nucleotide pool imbalance and replication stress (Techer et al., 2017). In the presence of PARGi, both Kuramochi and a subset of the transfected OVCAR3 cells became positive for γH2AX (Figure 4B). To focus on siRNAs that only elevated γH2AX in combination with PARG inhibition, we calculated the ratio of γH2AX in the presence and absence of PARGi (Figure 4C). This identified siRNAs targeting CHK1, MCM2, TIMELESS, HUS1, ATAD5, and RFC2, proteins involved in DNA replication and replication stress (Dungrawala et al., 2015). To validate these hits, we performed a secondary screen using independent pools of siRNAs in the same assay, revealing TIMELESS, HUS1, and RFC2 as the strongest hits (Figure 4D). Deconvolving the siRNA pools validated these targets as *bona fide* hits (Figure S4A), with all three inducing pan-nuclear γH2AX when combined with PARGi (Figures 4E and S4B–S4D). Moreover, inhibition of TIMELESS suppressed proliferation of OVCAR3 when combined with PARGi (Figure 4F). Inhibiting HUS1 and RFC2 also suppressed proliferation, albeit modestly (Figures S4C and S4D), consistent with them being weaker hits. The effect of inhibiting TIMELESS was not restricted to OVCAR3; the combination of PARGi and TIMELESS siRNA elevated γH2AX and suppressed proliferation in CAOV3, COV318, and COV362 (Figure S4E). Note that in the secondary screen, the CHK1 DDR checkpoint kinase manifested as a weaker hit because siCHK1 increased γH2AX in the absence of inhibitor (Figure S4B), consistent with inhibition of CHK1 being sufficient to induce replication stress (Syljuasen et al., 2005, Maya-Mendoza et al., 2007). Nevertheless, we conclude that the DNA replication factors TIMELESS, HUS1, and RFC2 are synthetic lethal with PARGi in OVCAR3. Also, because siRNAs targeting RPA1, RRM1, and, to a lesser extent, CHK1 induce the same pan-nuclear γH2AX phenotype, we conclude that the phenomenon induced by the PARGi in sensitive cells is indeed prolonged replication stress leading to replication catastrophe.

### PARG Inhibition Induces Replication Fork Asymmetry

**Figure 5:**
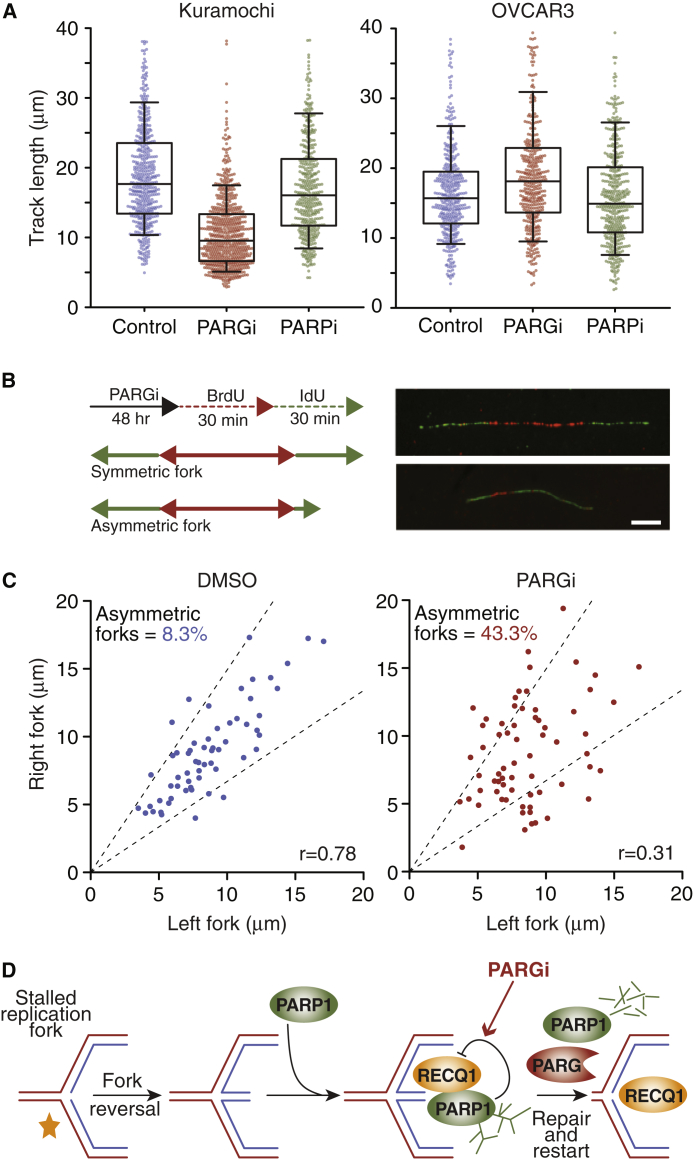
PARG Inhibition Causes Replication Fork Asymmetry (A) Measurement of DNA fibers following a 48 h exposure to 1 μM PARGi or 1 μM PARPi (n ≥ 400). Box-and-whisker plots show median, interquartile range, and 10%–90% range. (B) Experimental design and examples of symmetrical and asymmetrical DNA fibers. Scale bar, 10 μm. (C) Scatterplots of cognate left and right DNA fiber lengths in PARGi-treated Kuramochi cells, quantitating percentage of asymmetric forks and Spearman correlation (n > 60). Dashed lines indicate asymmetry cutoff defined as >30% difference between sister forks. (D) Schematic showing how PARGi reinforces PARP1-mediated inhibition of RECQ1, thereby suppressing fork restart (Berti et al., 2013). See also Figure S5.

Because siRNA-mediated inhibition of DNA replication factors induces PARGi sensitivity in OVCAR3, we reasoned that sensitive Kuramochi likely have an underlying DNA replication vulnerability that is exposed when PARG is inhibited. To test this, we pulsed cells with the nucleoside analog bromodeoxyuridine (BrdU) to analyze DNA replication fibers (Figure S5A). While addition of PARPi had little effect in either Kuramochi or OVCAR3, PARGi had a substantial effect in Kuramochi, reducing mean fiber length ∼1.7-fold (Figures 5A and S5A). To better define the nature of this defect, Kuramochi cells were sequentially pulsed with BrdU and iododeoxyuridine to measure replication fork symmetry (Figure 5B). Note that defects that cause replication fork stalling frequently lead to measurable asymmetry (Rodriguez-Lopez et al., 2002). In control cells, there was a good correlation between left and right fork length, but this was markedly reduced upon exposure to PARGi (Figures 5C and S5B). Thus, replication forks frequently stall and/or fail to restart when PARG is inhibited in Kuramochi cells. Because PARP1 stabilizes stalled replication forks by suppressing RECQ1-dependent fork restart (Bryant et al., 2009, Berti et al., 2013), we reasoned that by preventing PAR chain catabolism, the PARGi reinforces PARP1-mediated inhibition of RECQ1 (Figure 5D). Consistent with this notion, siRNA-mediated inhibition of RECQ1 in Kuramochi increased the number of γH2AX-positive cells, enhanced nuclear area, and suppressed proliferation (Figure S5C and data not shown). Moreover, RECQ1 siRNA sensitized Kuramochi cells to 50 nM PARGi, a concentration that had no effect on its own (Figure S5C). By contrast, RECQ1 siRNA had no obvious effect on OVCAR3 cells. Persistently stalled forks can eventually collapse and undergo nucleolytic degradation. Indeed, following prolonged PARGi exposure, we detected elevated DNA damage in a comet assay, increased RPA foci, an indicator of single-stranded DNA, and enhanced phosphorylation of RPA2 (Figures S5D–S5F). Thus, we conclude that Kuramochi cells have an underlying replication vulnerability that is heavily dependent on PARG activity to restart stalled replication forks; when PARG is inhibited, stalled forks fail to restart, leading to persistent replication stress that eventually leads to replication catastrophe.

### Interrogating DNA Replication Gene Expression Identifies Additional PARG Inhibitor Sensitive Lines

**Figure 6:**
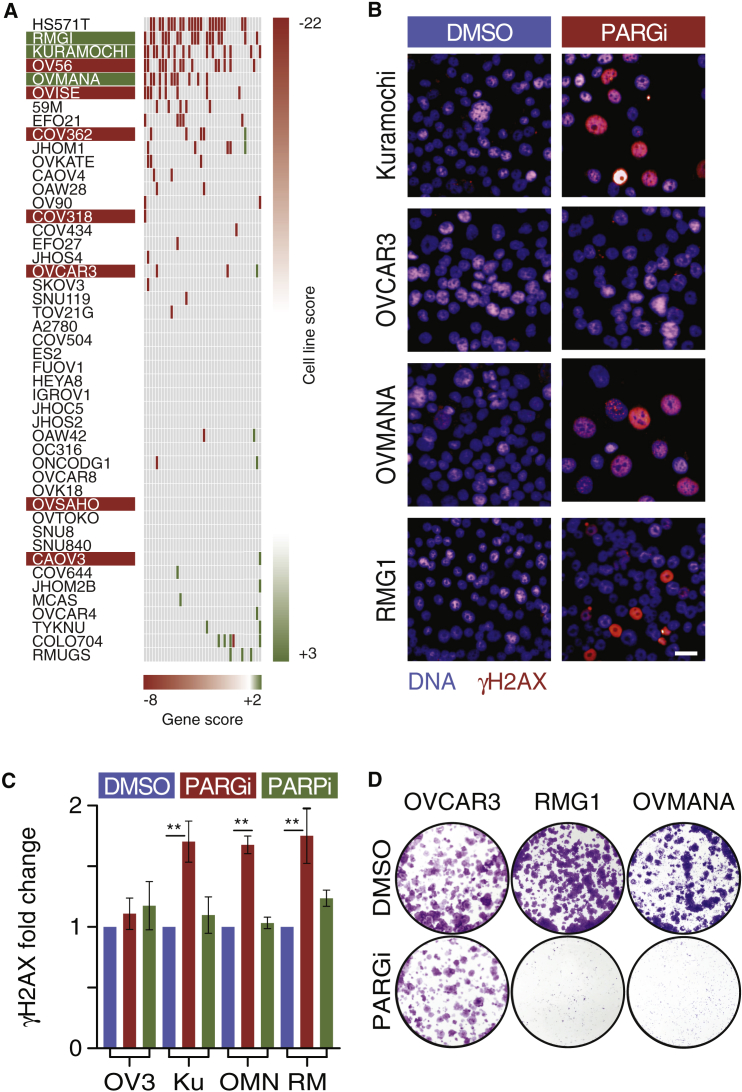
Interrogating DNA Replication Gene Expression Identifies Additional PARG Inhibitor Sensitive Lines (A) Heatmap showing upregulated (green) and downregulated (red) DNA replication genes in 47 ovarian cancer cell lines. (B) Immunofluorescence images of Kuramochi, OVCAR3, OVMANA, and RMG1 cells exposed to 1 μM PARGi for 48 h then stained to detect γH2AX. Scale bar, 10 μm. (C) Bar graph quantitating γH2AX, showing mean ± SEM derived from three independent experiments. ^∗∗^p < 0.01. (D) Colony formation in the continuous presence of 1 μM PARGi. See also Figure S6.

If an inherent DNA replication vulnerability accounts for Kuramochi sensitivity to PARGi, we reasoned that this might facilitate identification of other sensitive cell lines. To test this, we interrogated the expression of 40 core DNA replication genes in 47 ovarian cancer cell lines (Barretina et al., 2012). Notably, many of these genes were downregulated in six cell lines, including Kuramochi (Figures 6A and S6A). By contrast, very few if any were downregulated in OVCAR3 and the other four insensitive lines. Based on this, we set out to test whether HS571T, RMG1, OV56, OVMANA, and OVISE are also sensitive to PARGi. While HS571T cells are no longer available for research purposes, we sourced the other four lines, revealing that OVMANA and RMG1 are sensitive to PARGi; exposure to PARGi stabilized PAR chains, induced pan-nuclear γH2AX, and suppressed colony formation in both OVMANA and RMG1 (Figures 6B–6D, S1D, S6B, and S6C). By contrast, PARGi did not induce γH2AX or suppress proliferation in OV56 and OVISE (Figures S6B and S6C). This confirms that pan-nuclear γH2AX is a biomarker for PARGi sensitivity and shows that interrogating expression levels of DNA replication genes has the potential to predict PARGi sensitivity. In turn, this confirms that the underlying mechanism for PARGi sensitivity is indeed a DNA replication vulnerability. Note also that RMG1 and OVMANA are resistant to PARPi (Figures S6B and S6C), thus strengthening the notion that ovarian cancer cells show differential PARPi/PARGi sensitivity.

### Pharmacological Induction of Replication Stress Sensitizes Cells to PARG Inhibition

**Figure 7:**
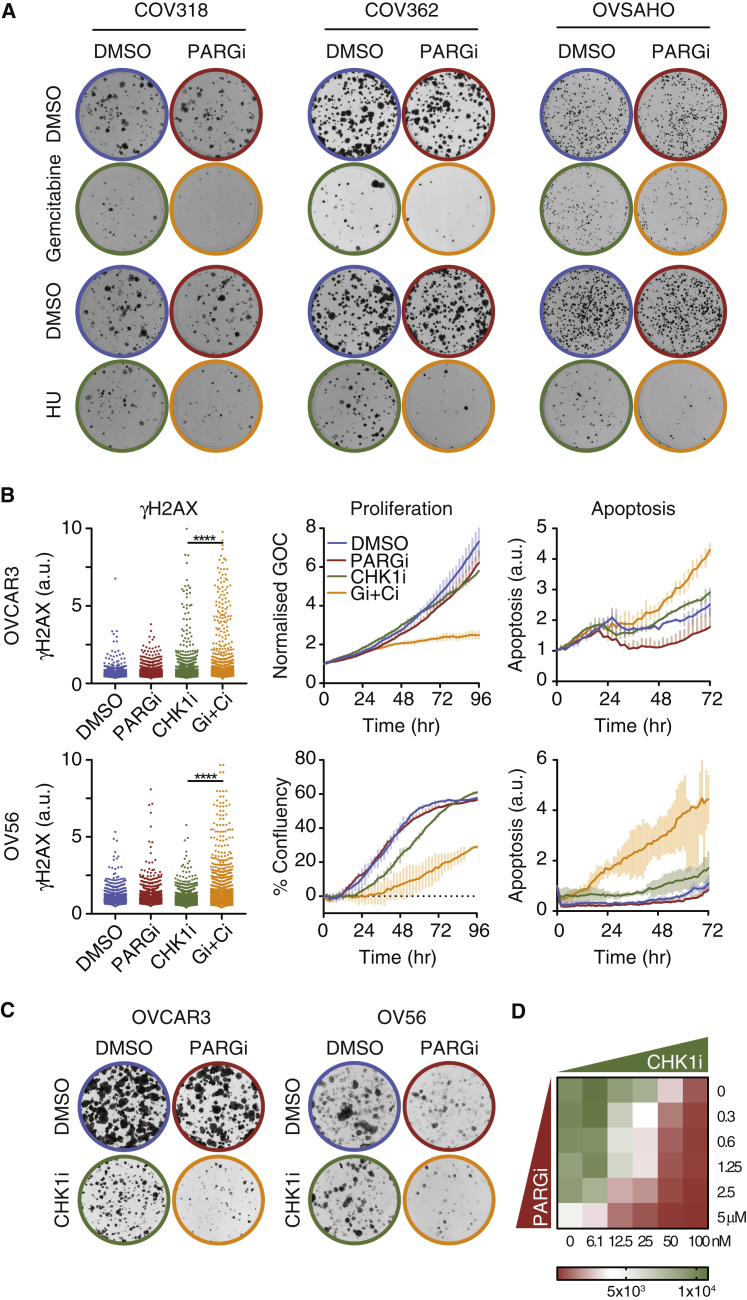
Replication Stress Sensitizes Cells to PARG Inhibition (A) Colony formation assays showing the combination effect of 1 μM PARGi and 4 nM gemcitabine or 200 μM hydroxyurea. Representative of three independent experiments. (B and C) γH2AX, proliferation (confluency), and apoptosis (caspase 3/7) (B) and colony formation (C) in response to PARGi plus CHK1i (75 nM for OV56; 50 nM and 25 nM for OVCAR3 in B and C, respectively). γH2AX values derived from at least 900 cells, and proliferation and apoptosis values show mean ± SD from two technical replicates. ^∗∗∗∗^p < 0.0001. All panels are representative of three independent experiments. (D) Heatmap measuring final cell number following a 96 h exposure to combinations of PARGi and CHK1i. See also Figure S7.

Of ten ovarian cancer cell lines tested thus far, three are sensitive to PARGi. To broaden the therapeutic potential of targeting PARG, we asked whether rational drug combinations could sensitize otherwise resistant lines. Because sensitive lines have an underlying DNA replication vulnerability and because PARGi is synthetic lethal with DNA replication genes in OVCAR3, we reasoned that inducing sub-lethal replication stress would sensitize cells to PARGi. Indeed, shRNA-mediated inhibition of PARG exacerbates replication stress induced by the topoisomerase inhibitor camptothecin and the RNR inhibitor hydroxyurea (Illuzzi et al., 2014, Ray Chaudhuri et al., 2015). Therefore, we tested PARGi in combination with gemcitabine, a nucleoside analog used to treat ovarian cancer. Of four lines tested, OVCAR3 were exquisitely sensitive to gemcitabine alone (data not shown) and PARGi had little influence on OVSAHO's sensitivity. By contrast, PARGi modestly sensitized COV318 and COV362 to gemcitabine (Figures 7A and S7A). The PARGi also modestly sensitized these lines and OVSAHO to hydroxyurea (Figures 7A and S7A). The PARGi also exacerbated the apoptosis-inducing effect of camptothecin on COV318 (Figure S7B). Thus, chemotherapy-induced replication stress can potentially sensitize additional ovarian cancer cells to PARG inhibition. However, because the effects were modest, we turned our attention to CHK1, which emerged from the synthetic lethality siRNA screen. Moreover, as a serine/threonine kinase, CHK1 is amenable to pharmacological inhibition. Indeed, CHK1 has attracted considerable attention as an oncology target, with several CHK1 inhibitors undergoing clinical evaluation (Garrett and Collins, 2011). Furthermore, several lines of evidence implicate PARG in the ATR/CHK1 pathway. In particular, PAR chains stabilize CHK1 binding at stalled replication forks, and depletion of PARG can activate ATR/CHK1 (Min et al., 2013, Ray Chaudhuri et al., 2015). To explore the effect of simultaneously inhibiting PARG and CHK1, we treated OVCAR3, COV318, and OV56 with PARGi in combination with the CHK1 inhibitor AZD7762, hereafter CHK1i. Notably, γH2AX and apoptosis became elevated while proliferation and colony formation were repressed (Figures 7B, 7C, S7C, and S7D). Indeed, concentration matrices indicated synergistic effects (Figures 7D and S7C). We conclude therefore that ovarian cancer cell lines not sensitive to PARGi can be sensitized when combined with a CHK1 inhibitor. Note that the CHK1i did not sensitize OV56 to PARPi (Figure S7E), further distinguishing these two modalities.

### Replication Stress Sensitizes Patient-Derived Ovarian Cancer Models to PARG Inhibition

**Figure 8:**
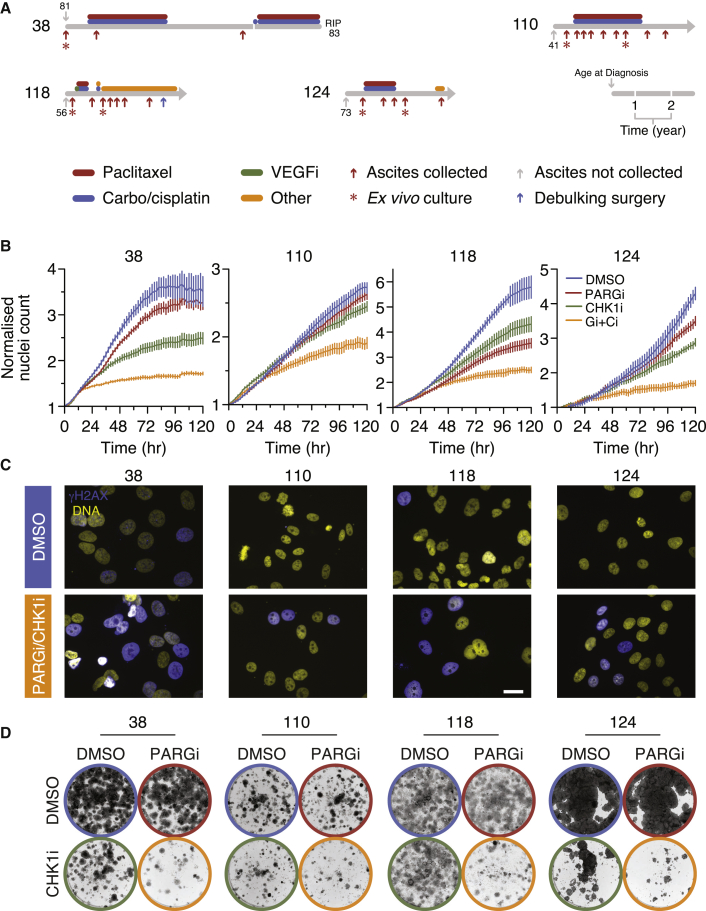
Inhibition of CHK1 Sensitizes Patient-Derived Ovarian Cancer Models to PARG Inhibition (A) Patient timelines showing age at diagnosis, chemotherapy treatments, and biopsy collections. (B) Proliferation curves derived by time-lapse imaging of the indicated models expressing a fluorescent protein-tagged histone, with nuclear count normalized to the value at T = 0. Values show mean ± SD from 12 technical replicates. Cells exposed to 1 μM PARGi plus 200 nM (38), 100 nM (110), 50 nM (118), and 50 nM (124) CHK1i. (C) Immunofluorescence images of cells treated as in (B) showing γH2AX following a 96 h exposure. Scale bar, 30 μm. (D) Colony formation assays of cells treated as in (B) for 96 h then fixed after 8 days. See also Figure S8.

While judiciously selected cell lines provide tractable models to study cancer cell biology, they massively underrepresent the genomic heterogeneity exhibited by primary cancers. Indeed, clonal evolution analysis of colorectal cancer cell lines shows that while all chromosomes are subject to segregation errors, karyotype evolution is highly constrained, leading to the persistence of a limited set of genomic configurations (Wangsa et al., 2018). By contrast, *ex vivo* cultures generated from ovarian cancer biopsies and analyzed at low passage display extensive genomic chaos and highly divergent karyotypes (our unpublished results). Therefore, to determine whether our observations based on established cell lines translated to a clinically relevant context, we tested PARGi on a panel of *ex vivo* ovarian cancer models (OCMs) (our unpublished results). Here, we focused on four models generated from chemo-naive ascites, OCMs 38, 110, 118, and 124 (Figure 8A and Table S1). Note that these models displayed the hallmarks of serous ovarian cancer, namely defective p53 responses and extensive aneuploidy (Figures S8A and S8B). As demonstrated by proliferation, pan-nuclear γH2AX, and colony formation, OCM.38 was insensitive to PARGi, partially sensitive to CHK1i, but particularly sensitive to the combination (Figures 8B–8D and S8C–S8E). By contrast, while both PARGi and CHK1i in isolation had only a marginal effect on OCM.110 and OCM.124, the combination was effective. OCM.118 was partially sensitive to both PARGi and CHK1i alone, but once again the combination enhanced sensitivity. Because overriding the G_2_/M checkpoint drives PARGi-arrested cells into aberrant mitoses (Figure S2B), we added WEE1i to the PARGi/CHK1i combination, and this further suppressed colony formation. Importantly, these drug combinations had no obvious effect on stromal fibroblasts or non-transformed fallopian tube epithelial cells (Figure S8E). Thus, we conclude that the mechanistic insight derived from the analysis of established cell lines does extend to clinically relevant models of ovarian cancer, and that PARG inhibitors open up opportunities to treat patients with ovarian cancer. Strikingly, patients 118 and 124 harbored low-grade serous tumors that did not respond to first-line carboplatin, indicating platinum-refractory disease (Table S1), a situation with very poor prognosis. Also, while the tumors in patients 38 and 110 initially responded, they quickly developed platinum resistance. Thus, PARG/CHK1 inhibitor combinations may have promise in both high-grade and low-grade serous ovarian cancers and may offer an early intervention for patients with platinum-refractory disease.

## Discussion

Here, we show that a subset of preclinical OCMs, including established cell lines and *ex vivo* cultures derived from patient biopsies, are sensitive to pharmacological inhibition of PARG. Furthermore, additional models can be rendered sensitive when combined with therapeutic agents that induce replication stress, in particular an inhibitor targeting the DNA damage response checkpoint kinase CHK1. Our observations indicate that sensitivity arises due to an underlying DNA replication vulnerability that renders cells dependent on PARG activity, such that upon PARG inhibition, stalled DNA replication forks fail to restart, leading to persistent replication stress. While vulnerable cells can survive transient suppression of PARG activity, prolonged inhibition likely leads to fork collapse, resulting in an accumulation of DNA damage, persistent ATM activation, replication catastrophe, and diminished clonogenic potential. Several observations support this interpretation; in particular, PARG inhibition induces (1) pan-nuclear γH2AX, a hallmark of replication catastrophe (Toledo et al., 2017); (2) a WEE1-dependent pre-mitotic cell-cycle block; (3) DNA replication fiber asymmetry, indicative of fork stalling (Merrick et al., 2004); and (4) DNA damage. Moreover, (5) PARG inhibition is synthetic lethal with inactivation of several DNA replication factors; (6) profiling the expression levels of DNA replication genes allowed us to identify additional, sensitive cell lines; and finally (7) cells not sensitive become dependent on PARG function in response to drug-induced replication stress.

Taken together with a number of previous reports, our observations support an emerging model whereby PARG counterbalances PARP1 to restart stalled replication forks. Firstly, while PARG-depleted cells can recover from transient replication stress induced by hydroxyurea, prolonged exposure results in fork collapse, double-strand breaks, and lethality (Illuzzi et al., 2014). Secondly, PARG depletion slows replication fork progression and leads to post-replicative single-stranded DNA gaps and reversed replication forks (Ray Chaudhuri et al., 2015). And finally, at telomeres, inhibiting PARG suppresses replication fork restart (Margalef et al., 2018). Coupled with PARP1's ability to stabilize stalled replication forks and suppress RECQ1-dependent fork restart (Bryant et al., 2009, Berti et al., 2013), one explanation for the underlying vulnerability responsible for PARG inhibitor sensitivity is frequent replication fork stalling. In unperturbed cells, this is rescued by PARP1-dependent fork stabilization and fork reversal, followed by RECQ1-dependent fork restart. Because the PARylation activity of PARP1 counteracts RECQ1 activity, restart requires PAR chain catabolism; thus, when PARG is inhibited, PAR chains persist, RECQ1 function is suppressed and fork restart is blocked (Figure 5D). Moreover, because reversed forks are particularly vulnerable to nucleolytic degradation, failure to restart inevitably leads to fork collapse (Mijic et al., 2017). While stalled replication forks are anticipated to activate the CHK1-dependent replication checkpoint, thereby restraining further origin firing in a cell-wide manner (Ge and Blow, 2010), an active checkpoint does not completely block S-phase progression: new origin clusters eventually activate despite the continued presence of replication stress, generating more aberrant forks and ultimately leading to replication catastrophe (Alvino et al., 2007, Petermann et al., 2010, Toledo et al., 2013). Consistently, while checkpoint activation is an early response to PARG inhibition, pan-nuclear γH2AX only emerges following prolonged exposure. A corollary of the lengthy delay before replication catastrophe occurs is that PARG inhibition does not compromise the ATR/CHK1-dependent checkpoint.

While this hypothesis has merit, why replication forks frequently stall in PARG inhibitor sensitive cells remains unclear. Understanding this will not only shed light on the mechanisms responsible for sensitivity but also aid development of predictive biomarkers. One possible reason for frequent fork collapse is deregulation of genes involved in DNA replication and/or replication stress responses, due to either oncogene-induced disruption of transcriptional networks (Bradner et al., 2017) or aneuploidy-induced gene dosage distortion (Oromendia and Amon, 2014). Consistent with this possibility, of the five ovarian cancer cell lines ranking highest for downregulation of DNA replication genes, three are sensitive to PARG inhibition, suggesting that a gene expression signature may have potential as a predictive biomarker. An important next step therefore will be a detailed interrogation of DNA replication pathways in an expanded panel of sensitive cell lines to derive more discerning biomarkers.

The PARG inhibitor synthetic lethal genes and several of the genes downregulated in sensitive cell lines, including *TIPIN*, *ATRIP*, *ATR*, and *RAD51*, are intimately implicated in maintaining replication fork integrity (Toledo et al., 2017, Liao et al., 2018). Following replication fork stalling, the heterotrimeric RPA complex is recruited to nascent single-stranded DNA. Chromatin-bound RPA, in concert with TIPIN and its obligate binding partner, TIMELESS, a PARGi synthetic lethal protein, recruits ATRIP and its binding partner, the ATR checkpoint kinase (Smith et al., 2009). Together with other constitutive replisome components, including the hexameric MCM2-7 complex and the RFC complex, the Rad9-HUS1-Rad1 (9-1-1) clamp loader is recruited to facilitate fork remodeling and replication checkpoint signaling via ATR/CHK1 (Techer et al., 2017). Note that *HUS1* and *MCM2* are PARG inhibitor synthetic lethal genes in OVCAR3, and that *RAD51* is downregulated in RMG1 and OVMANA, and the PARG inhibitor synthetic lethal genes *RFC2* and *ATAD5* encode components of the RFC complex (Ulrich, 2013). Because deregulating this network enhances fork stalling, this rationale provides a mechanistic explanation for PARG inhibitor sensitivity. Indeed, it is noteworthy that *RPA1-3* are downregulated in approximately 30% of HGSOC, and together *TIPIN*, *TIMELESS*, *ATRIP*, *ATR*, and *HUS1* are downregulated in 15% of cases (The Cancer Genome Atlas Research Network, 2011). Also, DNA fiber assays in short-term ovarian cancer cultures found that 61% displayed unstable replication forks (Hill et al., 2018). Moreover, *ATAD5* is a tumor suppressor; haploinsufficient mice display genomic instability and are cancer prone (Bell et al., 2011), and a genome-wide association study identified *ATAD5* as an ovarian cancer susceptibility locus (Kuchenbaecker et al., 2015). Interrogating this network is therefore a priority for defining a mechanism-derived predictive biomarker of PARG inhibitor sensitivity. It is possible that other vulnerabilities render cells dependent on PARG activity. Indeed, inactivation of BRCA1, BRCA2, PALB2, FAM175A, and BARD1 are synthetic lethal with PARG depletion in MCF7 breast cancer cells due to disruption of HR-mediated DDR (Gravells et al., 2017). Note, however, that while BRCA1 and BRCA2 are also involved in protecting stalled replication forks (Liao et al., 2018), they did not manifest as synthetic lethal targets in OVCAR3 cells. This suggests that PARG inhibitor sensitivity may be context dependent, in turn indicating that more comprehensive synthetic lethal screens in a wider range of cell lines and with different endpoints may also provide additional mechanistic insight, in turn aiding development of predictive biomarkers.

Despite an underlying DNA replication vulnerability, sensitive cells can resist brief periods of PARG inhibition. However, prolonged exposure diminishes viability, consistent with persistent replication stress inducing replication catastrophe (Toledo et al., 2017). We show that PARG-inhibitor-induced lethality is accelerated by WEE1 inhibition, driving arrested cells into catastrophic mitoses. This provides a compelling rationale for combining PARG and WEE1 inhibitors as a therapeutic strategy in ovarian cancer. Drugs targeting WEE1 are currently being investigated in clinical trials in a number of tumor types, as both monotherapies and in combination with DNA damaging agents (Matheson et al., 2016, Brandsma et al., 2017). Our observations suggest that WEE1 inhibition accelerates elimination of cells sensitive to PARG inhibition, providing a rationale for exploring PARG/WEE1 inhibitor combinations. Our observations also provide a compelling rationale for PARG/CHK1 inhibitor combinations. Originally developed as chemosensitizers and radiosensitizers due to CHK1's essential role maintaining genome integrity via WEE1 activation, CHK1 inhibitors also have potential as single agents to exploit cancer-cell-specific vulnerabilities (McNeely et al., 2014, Rundle et al., 2017). In particular, loss of G_1_/S control and oncogene-induced replication stress render cancer cells sensitive to CHK1 inhibitors (Murga et al., 2011). Consistently, several of the OCMs described here are sensitive to CHK1 inhibitors but importantly, this sensitivity was enhanced by PARG inhibition, opening up a wider therapeutic window, a window that might be further enhanced by also inhibiting WEE1. In light of the essential role ATR plays in activating CHK1 in response to replication stress (Burrows and Elledge, 2008), our observations also provide a strong case for exploring ATR/PARG inhibitor combinations. An important next step therefore will be systematic comparisons of PARG inhibition in combination with drugs targeting WEE1, CHK1, and ATR in a diverse panel of preclinical OCMs to determine whether any of these different pharmacological modalities offer distinct advantages.

A key issue is whether PARG inhibitors will offer distinct therapeutic opportunities compared with PARP inhibitors in the treatment of HGSOC. Our data show that these two modalities are indeed clearly differentiated, with several OCMs sensitive to one but not the other. Moreover, models sensitive to PARG/CHK1 inhibitor combinations were less affected by PARP/CHK1 inhibitor combinations, despite PARP1 being a multi-functional protector of stalled replication forks (Liao et al., 2018). This difference likely reflects the antagonistic roles played by these two enzymes in modulating PAR dynamics. Indeed, an shRNA screen in *Brca2*/*Trp53*-deficient mouse mammary tumor cells showed that PARG loss restored PARP1 signaling, leading to olaparib resistance (Gogola et al., 2018). Conversely, we show that olaparib protects HGSOC cells from PARG inhibition, consistent with the balance between these two activities controlling the response to replication stress, and consistent with PARG activity alleviating PARP1-mediated inhibition of fork restart. The net effect of disrupting PAR chain dynamics, favoring either synthesis or degradation, will likely depend on the DNA replication and repair vulnerabilities present in any given tumor cell. Thus, while PARG inhibitors may open up opportunities to treat PARP-inhibitor-resistant ovarian cancers, maximizing this opportunity will require robust, mechanism-derived predictive biomarkers.

PARG inhibitors suitable for clinical evaluation are not yet available. Our observations may, however, assist in the design of human trials testing clinical candidates, if and when they become available. In light of the excellent efficacy of PARP inhibitors in tumors harboring *BRCA1* or *BRCA2* mutations, and in light of the clear differences discussed above, efforts to test PARG inhibitors should focus on tumors considered unlikely to be PARP inhibitor responsive. Because sensitive OCMs harbor DNA replication vulnerabilities, “replication stress” enrichment biomarkers may facilitate identification of tumors most likely to respond to PARG inhibition, either alone or in combination with WEE1 inhibitors, while those deemed not to be sensitive are more likely to benefit from PARG/CHK1 inhibitor combinations. Because pan-nuclear γH2AX correlates with PARG inhibitor sensitivity, it represents a potential pharmacodynamic biomarker to monitor pathway modulation. Finally, because extensive cisplatin exposure can drive the emergence of multidrug resistance (Patch et al., 2015), early-phase clinical trials evaluating PARG inhibitors are more likely to succeed if they focus on patients early on in the treatment journey. Indeed, all the chemo-naive models tested here respond to the PARG/CHK1 inhibitor combination. Moreover, because these models were derived from patients with platinum-refractory/resistant disease, PARG/CHK1 inhibitor combinations may offer an alternative option for patients with an otherwise poor prognosis. Testing these concepts further in a diverse collection of well-characterized and clinically annotated OCMs will be important next steps.

## STAR★Methods

### Key Resources Table

**Table:** 

REAGENT or RESOURCE	SOURCE	IDENTIFIER
**Antibodies**		

Goat polyclonal anti-RFC2	Bethyl Laboratories	Cat# A300-142A; RRID: AB_155875
Rabbit monoclonal phopho-CHK1 (Ser345) (133D3)	Cell Signaling	Cat# 2348; RRID: AB_331212
Rabbit monoclonal anti-c-MYC (Y69)	Abcam	Cat# ab32072; RRID: AB_731658
Rabbit polyclonal anti-HUS1	Abcam	Cat# ab96297; RRID: AB_10680568
Rabbit monoclonal anti-LAMIN B1 (D9V6H)	Cell Signaling	Cat# 13435; RRID: AB_2737428
Rabbit polyclonal anti-phospho-RPA32 (S4/S8)	Bethyl Laboratories	Cat# A300-245A; RRID: AB_210547
Rabbit polyclonal anti-RPA32	Bethyl Laboratories	Cat# A300-244A; RRID: AB_185548
Rabbit monoclonal anti-RPA70	Abcam	Cat# ab79398; RRID: AB_1603759
Rabbit polyclonal anti-TIMELESS	Bethyl Laboratories	Cat# A300-961A; RRID: AB_805855
Sheep polyclonal anti-BUB3	(A.J. Holland and S.S.T., unpublished data)	N/A
Sheep polyclonal anti-PARG	This paper	N/A
Sheep polyclonal anti-TAO1	(Westhorpe et al., 2010)	N/A
Goat anti-mouse IgG (HL) HRP	Invitrogen	Cat# G21234; RRID: AB_2536530
Goat anti-rabbit IgG (HL) HRP	Merck Millipore	Cat# ABC240; RRID: AB_2722647
Rabbit anti-goat IgG (HL) HRP	Invitrogen	Cat# 81-1620; RRID: AB_2534006
Rabbit anti-sheep IgG (HL) HRP	Invitrogen	Cat# G21040; RRID: AB_2536527
Mouse monoclonal anti-p53 (DO-1)	Santa Cruz Biotechnology	Cat# sc-126; RRID: AB_628082
Mouse monoclonal anti-p21 (F-5)	Santa Cruz Biotechnology	Cat# sc-6246; RRID: AB_628073
Mouse anti-Poly ADP-ribose (Ab-1) (PAR)	Merck Millipore	Cat# AM80; RRID: AB_2155072
Mouse monoclonal anti-RECQL1 (RECQ1) (A-9)	Santa Cruz Biotechnology	Cat# sc-166388; RRID: AB_2178425
Mouse monoclonal anti-γH2AX (JBW301) (pS139)	Merck Millipore	Cat# 05-636; RRID: AB_309864
Rabbit polyclonal anti-γH2AX (pSer139)	Novus	Cat# NB100-384; RRID: AB_10002815
Rabbit monoclonal anti-Vimentin (EPR3776)	Abcam	Cat# ab92547; RRID: AB_10562134
Rabbit polyclonal anti-pKAP1 (S824)	Bethyl Laboratories	Cat# A300-767A; RRID: AB_669740
Rabbit polyclonal anti-RAD51	Bio Academia	Cat# 70-001; RRID: AB_2177110
Sheep polyclonal anti-CENP-F	(Hussein and Taylor, 2002)	N/A
Donkey anti-Mouse Cy2	Jackson ImmunoResearch Laboratories Inc	Cat# 715-225-150; RRID: AB_2340826
Donkey anti-Mouse Cy3	Jackson ImmunoResearch Laboratories Inc	Cat# 715-165-150; RRID: AB_2340813
Donkey anti-Rabbit Cy2	Jackson ImmunoResearch Laboratories Inc	Cat# 711-225-152; RRID: AB_2340612
Donkey anti-Rat Cy3	Jackson ImmunoResearch Laboratories Inc	Cat# 712-165-153; RRID: AB_2340667
Donkey anti-Sheep Cy5	Jackson ImmunoResearch Laboratories Inc	Cat# 713-175-147; RRID: AB_2340730
Mouse monoclonal anti-BrdU (B44)	BD Biosciences	Cat# 347580; RRID: AB_400326
Rat monoclonal anti-BrdU [BU1/75 (ICR1)]	Abcam	Cat# 6326; RRID: AB_305426

**Bacterial and Virus Strains**		

BL21 Competent Cells	New England BioLabs	Cat# C2527
XL1-Blue Competent Cells	Agilent Technologies	Cat# 200249

**Biological Samples**		

Patient samples	MCRC Biobank, Manchester	

**Chemicals, Peptides, and Recombinant Proteins**		

Aphidicolin	Sigma Aldrich	Cat#A4487
AZD1775 (WEE1i)	Selleckchem	Cat# S1525
AZD7762 (CHK1i)	Astra Zeneca	N/A
Bromodeoxyuridine (BrdU)	Sigma Aldrich	Cat# B5002
(S)-(+)-Camptothecin	Sigma Aldrich	Cat# C9911
Crystal Violet	Sigma Aldrich	Cat# C0775
Gemcitabine	Selleckchem	Cat# S1714
Hoechst 33258	Sigma Aldrich	Cat# B1155
Hydrocortisone	Sigma Aldrich	Cat# H0888
Hydroxyurea	Sigma Aldrich	Cat# H8627
Insulin	Sigma Aldrich	Cat# I9278
Iododeoxyuridine (IdU)	Sigma Aldrich	Cat# I7125
Olaparib (PARPi)	Selleckchem	Cat# S1060
PDD00017272 (PARGi)	(James et al., 2016)	N/A
PDD00031704 (PARGi-Me)	(James et al., 2016)	N/A
Polybrene	Merck Millipore	Cat# TR-1003-G
Propidium Iodide	Sigma Aldrich	Cat# P4170
Puromycin	Sigma Aldrich	Cat# P8833
RNase A	Thermo Scientific	Cat# EN0531
YOYO-1	Invitrogen	Cat# Y3601
17β Estradiol	Sigma Aldrich	Cat# E2758
Ascorbic acid	Sigma Aldrich	Cat# A5960
Bovine Serum Albumin (BSA)	Sigma Aldrich	Cat# A2153
Cholera toxin	Sigma Aldrich	Cat# C8052
Cholesterol	Sigma Aldrich	Cat# C3045
Choline chloride	Sigma Aldrich	Cat# C7527
EGF	Sigma Aldrich	Cat# E9644
Ergocalciferol	Sigma Aldrich	Cat# E5750
Folic acid	Sigma Aldrich	Cat# F8758
HEPES	Sigma Aldrich	Cat# H4034
Hydrocortisone	Sigma Aldrich	Cat# H0888
Hypoxanthine	Sigma Aldrich	Cat# H9636
i-Inositol	Sigma Aldrich	Cat# I75008
Insulin	Sigma Aldrich	Cat# I9278
L-Glutamine	Sigma Aldrich	Cat# 25030024
Lipoic acid	Sigma Aldrich	Cat# T1395
Medium 199 (10x)	Life Technologies	Cat# 11825015
Nutrient Mixture F12-Ham	Sigma Aldrich	Cat# N6760
O-phosphorylethanolamine	Sigma Aldrich	Cat# P0503
Para-aminobenzoic	Sigma Aldrich	Cat# A9878
Penicillin-Streptomycin	Sigma Aldrich	Cat# 15140122
Retinoic acid	Sigma Aldrich	Cat# R2625
Ribose	Sigma Aldrich	Cat# R9629
Selenious acid	Sigma Aldrich	Cat# 211176
Thamine HCL	Sigma Aldrich	Cat# T1270
α-tocopherol phosphate	Sigma Aldrich	Cat# T2020
Transferrin	Sigma Aldrich	Cat# T8158
Tridothyronine	Sigma Aldrich	Cat# T2877
Uracil	Sigma Aldrich	Cat# U1128
Vitamin B12	Sigma Aldrich	Cat# V6629
Xanthine	Sigma Aldrich	Cat# X4002

**Critical Commercial Assays**		

Click-iT™ Plus EdU Alexa Fluor™ 488 Imaging kit	Invitrogen	Cat# C10637
OxiSelect Comet Assay kit	Cell Biolabs, Inc.	Cat# STA-350
QIAprep® Spin Miniprep kit	Qiagen	Cat# 27106
Red blood cell lysis	Miltenyi Biotec	Cat# 130-094-183

**Deposited Data**		

scWGS karyotyping	European Nucleotide Archive, EMBL-EBI	ENA: PRJEB28664

**Experimental Models: Cell Lines**		

AAV293T	Agilent Technologies	Cat# 240073
CAOV3	American Type Culture Collection	Cat# ATCC HTB-75
COV318	Sigma Aldrich	Cat# 07071903
COV362	Sigma Aldrich	Cat# 07071910
Kuramochi	JCRB Cell Bank	Cat# JCRB0098
OVCAR3	American Type Culture Collection	Cat# ATCC HTB-161
OV56	Sigma Aldrich	Cat# 96020759
OVISE	JCRB Cell Bank	Cat# JCRB1043
OVMANA	JCRB Cell Bank	Cat# JCRB1045
OVSAHO	JCRB Cell Bank	Cat# JCRB1046
RMG1	JCRB Cell Bank	Cat# JCRB0172

**Oligonucleotides**		

Primer: *BamH*I PARG ATTAGGATCCATGAGCAGCGTGCAGAAGG	Invitrogen	N/A
Primer: *Not*I PARG TAATGCGGCCGCCTTGCTGTCTTTTTTGCC	Invitrogen	N/A
Primary screen siRNAs	Dharmacon/Horizon Discovery	Table S2
Secondary screen siRNAs	Dharmacon/Horizon Discovery	Table S2
Other siRNAs	Dharmacon/Horizon Discovery	Table S2

**Recombinant DNA**		

pGex-4T-3	GE Healthcare	Cat# 28-9545-52
pGEX-4T-3-PARG	This paper	N/A
pLVX-mCherry-N1	Takara Bio	Cat# 632562
pLVX-H2B-mCherry	This paper	N/A
pLVX-myc-GFP-H2B	This paper	N/A
psPAX2	A gift from Didier Trono (Addgene)	Cat# 12260
pMD2.G	A gift from Didier Trono (Addgene)	Cat# 12259

**Software and Algorithms**		

cBioPortal	(Cerami et al., 2012)	RRID: SCR_014555
ChemiDoc™ Touch Imaging System	Bio-Rad Laboratories	Cat# 1708370
CometScore 2.0	TriTek Corp.	http://rexhoover.com/index.php?id=cometscore
Columbus™ Image Data Storage and Anaylsis System	Perkin Elmer	Cat# Columbus
CoolSNAP HQ^2^ camera	Photometrics	N/A
Flowjo^©^	Flowjo, LLC	RRID: SCR_008520
Harmony High Content Imaging and Analysis Software	Perkin Elmer	Cat# HH17000001
Illustrator^®^ CC 2018	Adobe Systems Inc.	RRID: SCR_010279
ImageJ	National Institute of Health	RRID: SCR_003070
IncucyteZOOM^®^	Essen Bioscience	GUI=2016A
MetaMorph^®^ Microscopy Automation & Image Analysis Software	Molecular Devices	RRID: SCR_002368
Photoshop^®^ CC 2015	Adobe Systems Inc.	RRID: SCR_014198
Prism 7	GraphPad	RRID: SCR_002798
SeqMan Pro (Lasergene)	DNASTAR	RRID: SCR_000283
VisionWorks^®^ LS	UVP	N/A

**Other**		

6 well plates	Corning	Cat# 353046
25 cm^2^ flasks	Corning	Cat# 430639
75 cm^2^ flasks	Corning	Cat# 430641
Primaria™ 25 cm^2^ flasks	Corning	Cat# 353808
Primaria™ 75 cm^2^ flasks	Corning	Cat# 353810
96 well black μclear^®^ plates	Greiner Bio-One	Cat# 655087
96 well CellCarrier plates	Perkin Elmer	Cat# 6005550
Amintra Glutathione Resin	Expedeon	Cat# AGS0010
Bambanker™	Wako pure chemical ind. Ltd	Cat# 302-14681
Collagen Type 1 rat tail	Life Technologies	Cat# 354236
DharmaFECT 1	Dharmacon/Horizon Discovery	Cat# T-2001-03
DMEM/F-12 medium	Life Technologies	Cat# 11320074
Dulbecco’s Modified Eagle Medium (DMEM)	Life Technologies	Cat# 41966052
EZ-Chemiluminescence Detection Kit for HRP	Geneflow Limited	Cat# KI-0172
Fetal Bovine Serum Heat Inactivated	Life Technologies	Cat# F9665
H_2_O (molecular grade)	Merck Millipore	Cat# H2OMB0106
Ham’s F-12 Nutrient Mix medium	Life Technologies	Cat# 21765029
Hanks’ Balanced Salt Solution (HBSS)	Life Technologies	Cat# 14170088
Hyclone™ Fetal Bovine Serum	GE Healthcare	Cat# SH30070.03
Immobilon-P PVDF Membrane	Merck Millipore	Cat# IPVH00010
IncuCyte® Caspase 3/7 Green Apoptosis Reagent	Essen BioScience	Cat# 4440
IPTG	Bioline	Cat# BIO-37036
Luminata Forte Western HRP Substrate	Merck Millipore	Cat# WBLUF0100
NuPAGE™ 4-12% Bis-Tris protein gels (1.0 mm)	Life Technologies	Cat# NP0321BOX
Opti-MEM™	Life Technologies	Cat# 11058021
Platinum™ *Taq* DNA Polymerase	Invitrogen	Cat# 10033232
RPMI 1640	Life Technologies	Cat# 21875034
Superfrost Plus™ Adhesion Microscope Slides	Thermo Scientific	Cat# J1800AMNT

### Contact for Reagent and Resource Sharing

Further information and requests for resources and reagents should be directed to and will be fulfilled by the Lead Contact, Stephen S. Taylor (stephen.taylor@manchester.ac.uk).

### Experimental Model and Subject Details

#### Human Cell Lines

The human, female ovarian carcinoma cell lines COV318, COV362 (Sigma), CAOV3 (ATCC) were cultured in DMEM, while OVCAR3 (ATCC), Kuramochi, OVSAHO, OVMANA and OVISE (JCRB Cell Bank) were cultured in RPMI. RMG1 (JCRB Cell Bank) were cultured in Hams-F12 media. All cell lines were grown with 10% fetal bovine serum, 100 U/ml penicillin, 100 U/ml streptomycin and 2 mM glutamine and were maintained at 37°C in a humidified 5% CO_2_ atmosphere. OV56 (Sigma) were cultured in DMEM/F12 as above but supplemented with 10 μg/ml insulin, 0.5 μg/ml hydrocortisone and 5% fetal bovine serum. All lines were authenticated by the Molecular Biology Core Facility at the CRUK Manchester Institute using Promega Powerplex 21 System and periodically tested for mycoplasma.

#### OCMI Media

Nutrient Mixture F12-Hams: Medium 199 (50:50) mixed media containing 5% FBS (Life Science group) or 5% Hyclone™ FBS (GE Healthcare); 2 mM glutamine; 100 U/ml penicillin; 100 U/ml streptomycin; 10 mM HEPES at pH 7.4; 20 μg/ml insulin; 0.01 μg/ml EGF; 0.5 μg/ml hydrocortisone; 10 μg/ml transferrin; 0.2 pg/ml Tridothyronine; 5 μg/ml o-phosphoryl ethanolamine; 8 ng/ml selenious acid; 0.5 ng/ml 17β-oestradiol; 5 μg/ml all trans retinoic acid; 1.75 μg/ml hypoxanthine; 0.05 μg/ml lipoic acid; 0.05 μg/ml cholesterol; 0.012 μg/ml ascorbic acid; 0.003 μg/ml α-tocopherol phosphate; 0.025 μg/ml calciferol; 3.5 μg/ml choline chloride; 0.33 μg/ml folic acid; 0.35 μg/ml vitamin B12; 0.08 μg/ml thamine HCL; 4.5 μg/ml i-inositol; 0.075 μg/ml uracil; 0.125 μg/ml ribose; 0.0125 μg/ml para-aminobenzioic acid; 1.25 mg/ml BSA; 0.085 μg/ml xanthine and 25 ng/ml cholera toxin (all from Sigma).

#### Patient Sample Collection

Research samples were obtained from the Manchester Cancer Research Centre (MCRC) Biobank, UK. The role of the MCRC Biobank is to distribute samples and therefore cannot endorse studies performed or the interpretation of results. The MCRC Biobank is licensed by the Human Tissue Authority (license number: 30004) and has been ethically approved as a research tissue bank by the South Manchester Research Ethics Committee (Ref: 07/H1003/161+5). A suite of standard Patient Information Sheets and Patient Consent Forms have been developed and approved and informed patient consent was obtained before patient samples were taken. Please refer to the following website for more information: www.mcrc.manchester.ac.uk/Biobank/Ethics-and-Licensing.

#### Establishment of *Ex Vivo* Models

Chemo-naïve ascitic fluid was centrifuged at 500xg for 10 min at 4°C and cell pellets pooled in HBSS media. Red blood cells were removed using a red blood cell lysis buffer (Miltenyi Biotec) as per the manufacturer’s instructions. Tumor cells were counted and plated at 1 million cells in two collagen-coated 75 cm^2^ flasks and cultured in OCMI media containing 5% Hyclone™ FBS (GE Healthcare). All cultures were initially incubated for 2-4 day at 37°C in a humidified 5% CO_2_, 5% O_2_ atmosphere and media was replaced every 3-4 day. Upon cell attachment, stromal cells were separated from the mixed sample using 0.05% trypsin-EDTA and plated in gelatin-coated 75 cm^2^ flasks in OCMI media containing 5% FBS. Once tumor cells reach 95% confluency, cells were passaged using 0.25% Trypsin-EDTA, centrifuged in DMEM containing 20% FBS and re-plated at a 1:2 ratio. For long-term storage, cells were frozen in Bambanker™ (Wako pure chemical).

### Method Details

#### Materials and Plasmids

The PARG inhibitor, PDD00017272 (PARGi), and an inactive methylated analogue, PDD00031704 (PARGi-Me) (James et al., 2016), were dissolved in DMSO and used at a final concentration of 1 μM unless otherwise indicated. The CHK1 inhibitor, AZD7762 (CHK1i), was dissolved in DMSO and used at final concentration as described in the figures; the PARP1/2 inhibitor, Olaparib (PARPi), was dissolved in DMSO and used at a final concentration of 1 μM; the WEE1 inhibitor, AZD1775 (WEE1i), was dissolved in DMSO and used at a final concentration of 200 nM (all from Selleckchem). Camptothecin (Sigma) was dissolved in DMSO and used as indicated in the figures. Gemcitabine (Selleckchem) and hydroxyurea (Sigma) were dissolved in water and used at a final concentration as described in the figures. Aphidicolin (Sigma) was used at a final concentration of 0.58 μM. BrdU and IdU (Sigma) were dissolved in culture media and used at concentrations described in DNA fiber assay methods.

#### Flow Cytometry

For DNA content analysis, cells treated as indicated were harvested, washed in PBS, fixed in ice-cold 70% ethanol/PBS and stored -20°C overnight. Cells were then washed twice in PBS and stained with propidium iodide (40 μg/ml) (Sigma) and RNase A (50 μg/ml) (Thermo Scientific) for 30 min at room temperature. Post-staining, cells were stored at 4°C prior to analysis. For EdU staining the cells were labelled using the Click-iT Plus Edu Imaging kit (Invitrogen) according to the manufacturer’s instructions.

#### Immunofluorescence

Cell lines were plated onto 13 mm coverslips 24 hr prior to drug treatment. For the RMG1 cell line and the *ex vivo* models, the coverslips were pre-coated with collagen. Cells were washed and fixed in 1% formaldehyde, quenched in glycine, then incubated with primary antibodies (CENP-F 1:1000; γH2AX 1:2000; pKAP1 1:1000; PAR 1:2000; RAD51 1:1000; RPA70 1:500; Vimentin 1:1000) for 30 min at room temperature. Coverslips were washed two times in PBS-T (PBS, 0.1% Triton X-100) and incubated with the appropriate fluorescently conjugated secondary antibodies (1:500) for 30 min at room temperature. Coverslips were washed in PBS-T and DNA stained for 1 min with 1 μg/ml Hoechst 33258 (Sigma) at room temperature. Coverslips were further washed in PBS-T and mounted (90% glycerol, 20 mM Tris, pH 9.2) onto slides. Slides were stored at -20°C prior to image acquisition using an Axioskop2 (Zeiss, Inc.) microscope fitted with a CoolSNAP HQ camera (Photometrics) using MetaMorph Software (Molecular Devices). Image analysis was conducted using Adobe Photoshop^®^ CC 2015 (Adobe Systems Inc.). For high-throughput immunofluorescence, cells were processed as above in a 96 well plate format (PerkinElmer Cell Carrier plates) and stored in PBS at 4°C prior to imaging. Images were acquired using Operetta^®^ High Content Imaging System (Perkin Elmer), and quantified using Harmony and Columbus High Content Imaging and Analysis Software (Perkin Elmer) to measure fluorescence intensity or foci number, the latter using the Spot Finder tool.

#### Immunoblotting

Proteins were extracted by boiling cell pellets in sample buffer (0.35 M Tris pH 6.8, 0.1 g/ml sodium dodecyl sulphate, 93 mg/ml dithiothreitol, 30% glycerol, 50 μg/ml bromophenol blue), resolved by SDS-PAGE, then electroblotted onto Immobilon-P membranes (Merck Millipore). Following blocking in 5% dried skimmed milk (Marvel) dissolved in TBS-T (50 mM Tris pH 7.6, 150 mM NaCl, 0.1% Tween-20), membranes were incubated with primary antibodies (BUB3 1:1000; HUS1 1:500; LAMIN B1 1:1000; PARG 1:1000; c-MYC 1:3500; TP53 1:1000; P21 1:100; RFC2 1:1000; RECQL1 1:1000; RPA32 1:1000; p-RPA32 1:500; TAO1 1:1000; TIMELESS 1:1000) overnight at 4°C. Membranes were then washed three times in TBS-T and incubated for at least 1 hr with appropriate horseradish-peroxidase-conjugated secondary antibodies (1:2000). After washing in TBS-T, bound secondary antibodies were detected using either EZ-Chemiluminescence Reagent (Geneflow Ltd) or Luminata™ Forte Western HRP Substrate (Merck Millipore) and a Biospectrum 500 imaging system (UVP) or ChemiDoc™ Touch Imaging System (BioRad).

#### Lentiviral Production and Transduction

AAV293T cells were plated at 5x10^4^ cells per well in a 24 well plate. Media was replenished 1 hr before transfection. Cells were transfected with pLVX-mCherry-N1-based lentiviral plasmids (Takara Bio), modified to express human histone H2B tagged either at the N-terminus with GFP (pLVX-myc-EmGFP-H2B) or at the C-terminus with mCherry (pLVX-H2B-mCherry), and psPAX2 and pMD2.G (gifts from Didier Trono, Addgene) using 16.6 mM CaCl_2_ in DMEM supplemented with 10% Hyclone™ serum (GE Healthcare) and incubated overnight. Virus was harvested 48 hr after transfection, centrifuged and filtered (0.45 μm). Cells were seeded at 2-6 x10^5^ cells per well in a 12 well plate. 48 hr later, diluted lentivirus and 10 μg/ml polybrene was added to the cells. The plates were centrifuged at 300xg at 30°C for 2.5 hr. 1 ml of culture media was added and the plates incubated overnight. Puromycin (1 μg/ml) was added 48 hr post-transduction.

#### Drug Sensitivity Assay and Cell Fate Profiling

Cells were seeded at 1-2 x10^4^ cells per ml in a 96 well plate (Greiner Bio-One/ PerkinElmer Cell Carrier), 24 hr prior to drug treatment. For the RMG1 cell line and for the *ex vivo* models the plates were pre-coated with collagen. Cells were imaged using an IncuCyte^®^ ZOOM (Essen BioScience) equipped with a 20x objective and maintained at 37°C in a humidified 5% CO_2_ atmosphere or a 5% CO_2_ and 5% O_2_ atmosphere for the *ex vivo* models. Nine phase contrast and fluorescence images per well (for GFP-H2B or H2B-mCherry expressing cells) were collected every 1-6 hr when analyzing proliferation and drug sensitivity or every 10 min for cell fate profiling. IncuCyte^®^ ZOOM software was used in real-time to measure confluence and fluorescent object count. To measure apoptosis, cells were labeled with either propidium iodide (30 μM) or IncuCyte™ Caspase-3/7 Green Apoptosis Reagent (Essen Bioscience) and the number of fluorescent objects was calculated. For cell fate profiling, image sequences were exported in MPEG-4 format and analyzed manually to generate cell fate profiles. Timing data was imported into Prism 7 (GraphPad) for statistical analysis and presentation.

#### Colony Formation Assay

For colony formation assays either 500 or 1000 cells per well were seeded into 6 well plates (note, for RMG1 cells and for the *ex vivo* models the plates were pre-coated with collagen) and were either treated continuously with the inhibitors or the inhibitors were washed out at the specific time points as indicated in the figures. Once colonies had developed the cells were fixed in 1% formaldehyde and stained with 0.05% (w/v) crystal violet solution. Plates were then imaged using a ChemiDoc™ Touch Imaging System (BioRad).

#### Antibody Production

A cDNA fragment of the PARG gene encoding amino acids 109-408 was amplified using Platinum™ *Taq* DNA polymerase (Invitrogen) and the primers, *BamH*I PARG and *Not*I PARG. This fragment was ligated into a pGex-4T-3 vector (GE Healthcare) to produce the pGex-4T-3-PARG plasmid. The GST-PARG fusion protein was expressed in *E.coli* BL21 cells using 1 mM IPTG whilst shaking at 37°C for 2 hr. GST-PARG was purified using glutathione sepharose beads (Expedeon) and was used for sheep immunization (Orygen Antibodies Ltd). Polyclonal sheep anti-PARG antibodies were purified from generated serum by affinity purification according to standard procedures. In brief, serum was first passed through covalently coupled GST-glutathione sepharose beads to remove anti-GST antibodies. Covalently coupled GST-PARG beads were incubated with the serum, bound anti-PARG antibodies were eluted with 100 mM glycine (at pH 2.8), neutralized using 1 M sodium phosphate (pH 8.0), and dialyzed against PBS. Purified antibodies were finally run through GST-coupled sepharose beads to remove any remaining anti-GST antibodies.

#### siRNA Screen

##### Primary Screen

OVCAR3 (ATCC) cells were seeded at a starting density of 3000 cells per well into black, clear bottomed 96 well plates (Perkin Elmer, Cell Carrier) and were reverse transfected with siRNA SMARTpools consisting of 4 individual siGENOME oligonucleotides for each of the genes listed in Table S2 (Dharmacon/Horizon Discovery). Reverse transfection was carried out using Opti-MEM media, DharmaFECT-1 transfection reagent (Dharmacon/Horizon Discovery) and siRNAs at a final concentration of 66 nM, according to manufacturer’s instructions. 48 hr post-transfection, cells were treated with either DMSO or 1 μM PARGi for 48 hr. The cells were fixed and stained for γH2AX according to the immunofluorescence protocol and stored in PBS at 4°C prior to imaging using an Operetta® High Content Imaging System (Perkin Elmer). To account for plate effects and replicate variation, each plate contained the relevant control wells: transfection controls (non-targeting, mock targeting), negative controls (OVCAR3 cells, untreated and PARGi-treated) and positive controls (Kuramochi cells, untreated and PARGi-treated).

##### Secondary Screen

ON-TARGETplus siRNA SMARTpool consisting of four individual oligonucleotides, distinct from those used in the primary screen, were chosen based on genes identified in the primary screen (Table S2). Each well (DMSO and PARGi-treated) was transfected in duplicate. BRCA1 and BRCA2 siRNAs were also used as negative controls. The transfection and drug treatment protocol, and measurement of γH2AX fluorescence intensity followed the same procedure used for the primary screen.

##### siRNA Deconvolution

Four ON-TARGETplus siRNAs of the SMARTpool used in the secondary screen were deconvolved and transfected individually into OVCAR3 cells in the presence and absence of PARGi; the same procedure was used as the primary and secondary screens. siRNAs which displayed significant increase in γH2AX for n=3 experiments were chosen to re-pool for further assays. Note for HUS1, the individual siRNAs only had a modest effect so all four were re-pooled.

##### Data Analysis

For both primary and secondary screening, images were analyzed using Harmony High Content Imaging and Analysis Software (Perkin Elmer) to measure mean nuclear fluorescence intensity. Raw mean nuclear fluorescence intensity values were taken as an average per well and analyzed as follows:

Step 1: Normalization to the average of transfection controls to account for variation between replicates. This yields the values used in Figures 4B and S4B.
Step 2: Calculation of the ratio between normalized PARGi/DMSO values. This yields the values used in Figures 4C and 4D.
Step 3: Normalize to a given OVCAR3 negative control PARGi/DMSO ratio to account for variation between plates. This yields the values used in statistical analysis.
Step 4: Statistical analysis using GraphPad Prism 7 Software, using ANOVA (primary screen) and Kruskal-Wallis non-parametric test (secondary screen and siRNA deconvolution).

#### Proliferation Assay

Cell lines expressing GFP-H2B were transfected with the re-pooled custom siRNA SMARTpool and non-targeting siRNA according to the protocol used in the primary screen. 48 hr post-transfection cells were treated with DMSO or PARGi and imaged by time-lapse microscopy, taking 9 phase contrast and fluorescence images per well every 1-4 hr for a total of 96 hr. IncuCyte^®^ ZOOM software (Essen Bioscience) was used in real-time to measure confluence and green fluorescent object count. After imaging for 96 hr, cells were fixed and stained according to the immunofluorescence protocol and imaged using the Operetta® High Content Imaging System to give matching γH2AX fluorescence intensity measurements.

#### DNA Fiber Assay

Asynchronous cells were incubated in the presence of inhibitors for 48 hr then pulsed with media containing nucleotide analogue plus drug treatment and incubated under the same conditions. For single labelling, sub-confluent cells were pulsed with BrdU (Sigma) dissolved in media at a final concentration of 5 μM for 30 min followed by washing twice with ice-cold PBS. For double labelling, cells were first incubated with BrdU at 5 μM for 15 min, followed by incubating with IdU (Sigma) at 200 μM for 15 min. Following trypsinization, cells were diluted in ice-cold PBS to give a final concentration of 5x10^5^ cells per ml and kept on ice. The cell suspension (2 μl) was then dropped onto microscope slides and dried at room temperature for 5-10 min before adding 7 μl of spreading buffer (200 mM Tris-HCl pH 7.5, 50 mM EDTA, and 0.5% SDS), mixing gently and incubating for a further 5 min. Slides were tilted approximately 10-20° so that the drop runs across the length of the slide. Spread fibers were air dried and fixed in methanol/acetic acid (3:1) for 10 min, dried and stored at 4°C for a minimum of 24 hr. Slides were then washed 2 x H_2_O for 5 min, 1 x 2.5 M HCl, denatured with 2.5 M HCl for 1hr, rinsed 2 x PBS and then washed with blocking solution (PBS with 1% BSA and 0.1% Tween-20) for 2 x 5 min and 1 x 1 hr. For immuno-labelling all antibodies were dissolved in blocking solution. Slides were then incubated with a rat anti-BrdU antibody (Abcam) to detect BrdU (1:500) for 1 hr under humidified conditions, rinsed 3 x PBS, fixed for 10 min with 1% formaldehyde, rinsed 3 x PBS, and quenched in glycine. Slides were then rinsed 3 x PBS, 3 x 5 min washes with blocking solution and incubated overnight in the appropriate fluorescently conjugated secondary antibody (1:500) at 4°C for single labelling, or for 1.5-2 hr at room temperature for double labelling. For single labelling, slides were washed 2 x PBS, 3 x 5 min washes with blocking solution, 2 x PBS, before finally counterstaining with YOYO-1 (1:10,000 in PBS; Invitrogen) for 10 min, followed by further washing in PBS. For double labelling, slides were washed 2 x PBS, followed by overnight, 4°C incubation in primary anti-BrdU antibody (BD Biosciences) to detect IdU (1:100). Slides were then washed 2 x PBS, 3 x 5 min blocking solution, followed by incubation in the appropriate fluorescently conjugated secondary antibody (1:500) for 1.5 hr. Post-incubation, slides were washed 2 x PBS, 3 x 5 min with blocking solution and 2 x PBS. All slides were mounted to coverslips using PBS/Glycerol (1:1). Images were acquired using an Axioskop2 (Zeiss, Inc.) microscope fitted with a CoolSNAP HQ camera (Photometrics) and 2-5 slides analyzed per condition. Fiber lengths were quantified using ImageJ software (NIH).

#### Comet Assay

Comet assays were performed using an OxiSelect Comet Assay Kit (Cell BioLabs. Inc.), following manufacturer’s instructions. In brief, Kuramochi cells were treated with 1 μM PARGi for 72 hr prior to performing the experimental procedure. Post-72 hr, OxiSelect Comet Agarose was heated at 90°C in a water bath for 20 min then cooled in a 37°C water bath. Agarose was spread over each well of a slide to form a base layer and set at 4°C. Whilst setting, adherent cells were removed from the flask by scraping with a cell scraper, centrifuged at 700xg for 2 min, washed in ice-cold PBS (without Mg^2+^ and Ca^2+^), centrifuged and resuspended at 1x10^5^ cells per ml in ice-cold PBS (without Mg^2+^ and Ca^2+^). In parallel, control cells were prepared as above and were either placed immediately on ice or irradiated at 2 Gy using a Faxitron Irradiator; the latter control were placed on ice post-irradiation. Under low lighting, samples were combined with molten Comet Agarose at a 1:10 ratio (v/v), mixed by pipetting and immediately placed and spread on top of the pre-coated well. Maintaining the slide horizontally, slides were set in the dark at 4°C. Slides were immersed into pre-chilled lysis buffer (2.5 M NaCl, 100 mM EDTA, 1 x OxiSelect Comet Lysis buffer, pH 10.0) in the dark at 4°C for 1 hr and then replaced with pre-chilled alkaline solution (0.3 M NaOH, 1 mM EDTA) and incubated in the dark at 4°C for 30 min. Slides were transferred to a horizontal electrophoresis chamber and filled with cold alkaline electrophoresis buffer (300 mM NaOH pH >13, 1 mM EDTA). Voltage was applied at 1 volt/cm for 25 min; note that the buffer volume was adjusted to produce a current setting of 300 mA. The slides were immersed 3 x 2 min in pre-chilled dH_2_O before submerging in pre-chilled 70% ethanol for 5 min and allowed to air dry. Once dry, Vista Green DNA Dye was added to each well and incubated at room temperature for 15 min. Images were acquired using an EVOS Auto II cell imaging system (Invitrogen) and image analysis was conducted using CometScore 2.0 (TriTek Corp.).

#### BrdU Proliferation Assay

Cells were plated onto glass coverslips and treated for 48 hr with 2% DMSO followed by 48 hr washout or a further 48 hr treatment. Cells were pulsed with 10 μM BrdU in media with or without DMSO treatment for 1 hr, followed by ice-cold methanol fixation. Coverslips were incubated with 2.5 M HCl for 45 min, neutralized with 0.1 M sodium borate and washed 3 x PBS then 3 x PBS+ (PBS, 1% BSA, 0.1% Tween-20). Coverslips were then incubated in anti-BrdU antibody (1:500; Abcam) diluted in PBS+ for 30 min, washed in PBS+ and incubated in secondary anti-rat Cy3 antibody (1:500) diluted in PBS+ for a further 30 min. DNA was counterstained using YOYO-1 (Invitrogen) and coverslips mounted onto slides (90% glycerol, 20 mM Tris, pH 9.2). Images were acquired using an Axioskop2 (Zeiss, Inc.) microscope fitted with a CoolSNAP HQ camera (Photometrics) using MetaMorph Software (Molecular Devices).

#### scWGS-Based Karyotyping

Single G1 nuclei were isolated, sorted, and sequenced as described (Bakker et al., 2016, van den Bos et al., 2016). Briefly, cells were incubated in a cytoplasmic lysis buffer and stained with propidium iodide (10 μg/ml) and Hoechst 33258 (10 μg/ml). Single G1 nuclei were sorted in 96 well plates and stored at -80°C. Illumina-based library preparation was performed on a Bravo Automated Liquid Handling Platform (Agilent Technologies). Samples were sequenced on an Illumina NextSeq 450 at ERIBA (Illumina). Unprocessed sequencing reads were demultiplexed using library-specific barcodes and converted into fastq format using standard Illumina software (bcl2fastq version 1.8.4). Demultiplexed reads were aligned to human reference genome GRCh38 using Bowtie2 (version 2.2.4). Duplicate reads were marked and removed using BamUtil (version 1.0.3.). Aligned sequencing reads were analyzed and curated using AneuFinder (version 1.4.0, Bakker et al., 2016) using 1Mb bins.

### Quantification and Statistical Analysis

Prism 7 (GraphPad) was used for statistical analysis, where ^∗^ p < 0.05, ^∗∗^ p < 0.01, ^∗∗∗^ p < 0.001, ^∗∗∗∗^ p < 0.0001, ns: p > 0.05. Details of statistical analyses are described in the figure legends. Values on apoptosis and proliferation line graphs show the mean and SD or SEM from three technical replicates. Box-and-whisker plots show the median, interquartile ranges and the full range.

#### Data and Software Availability

The accession number for the scWGS-based karyotyping data reported in this paper is ENA: PRJEB28664.

## Supplemental Information

Document S1. Figures S1–S8 and Table S1

Table S2. List of siRNA Sequences, Related to STAR Methods

Document S2. Article plus Supplemental Information
