# Supplementary material for: DNA Replication Vulnerabilities Render Ovarian Cancer Cells Sensitive to Poly(ADP-Ribose) Glycohydrolase Inhibitors
Source: Cancer Cell. 2019 Mar 18;35(3):519–533.e8. doi: 10.1016/j.ccell.2019.02.004 (PMC6428690; doi:10.1016/j.ccell.2019.02.004)
Supplement: Document S1. Figures S1–S8 and Table S1 [file mmc1.pdf]

Cancer Cell, Volume *35*

Supplemental Information

DNA Replication Vulnerabilities

Render Ovarian Cancer Cells Sensitive

to Poly(ADP-Ribose) Glycohydrolase Inhibitors

Nisha Pillay, Anthony Tighe, Louisa Nelson, Samantha Littler, Camilla Coulson-Gilmer, Nourdine Bah, Anya Golder, Bjorn Bakker, Diana C.J. Spierings, Dominic I. James, Kate M. Smith, Allan M. Jordan, Robert D. Morgan, Donald J. Ogilvie, Floris Foijer, Dean A. Jackson, and Stephen S. Taylor

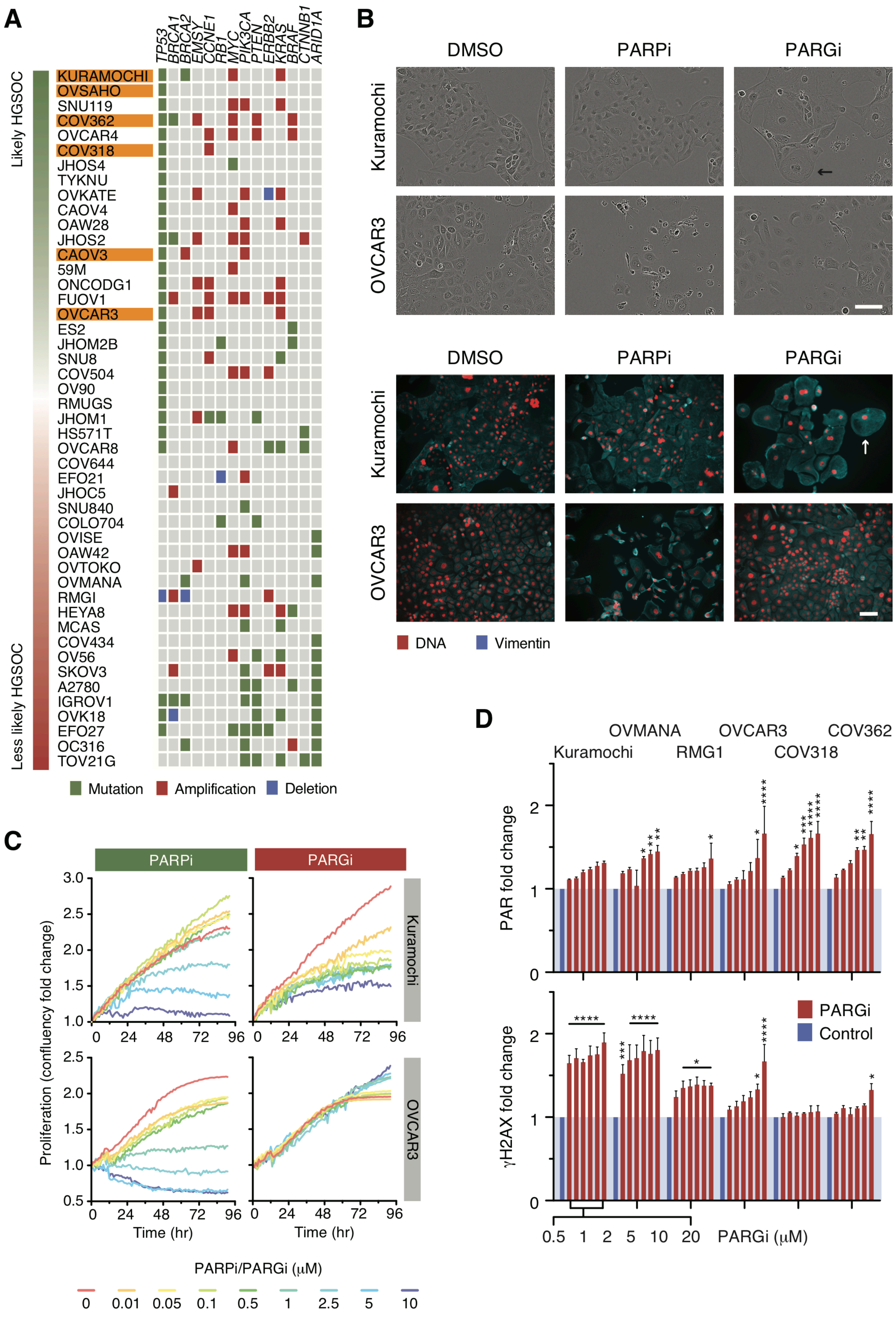

Figure S1, related to Figure 1. Ovarian cancer cells display differential sensitivity to PARG and PARP inhibitors. (A) List of 47 ovarian cancer cell lines analyzed by the Broad-Novartis Cancer Cell Line Encyclopedia (CCLE) showing mutational status of genes implicated in HGSOC and other types of ovarian cancer (Barretina et al., 2012). The list is rank ordered with cell lines likely reflecting HGSOC at the top (Domcke et al., 2013). The six lines selected for the PARGi screen shown in Figure 1 are highlighted in orange. Note that Figure 1A is based on (Domcke et al., 2013) while Figure S1A is based on updated data from cBioPortal Version 1.15.1 (Cerami et al., 2012). (**B**) Phase contrast and immunofluorescence images of

Kuramochi and OVCAR3 cells exposed to either 1 μM PARGi or 1 μM PARPi for 72 hr; scale bar 100 μm. Note that in the presence of PARGi, Kuramochi cells often adopt a “fried egg” appearance (see arrows), i.e. large, round cells with enlarged nuclei. (**C**) Proliferation curves measuring confluency fold change over 96 hr following exposure to PARPi and PARGi at the indicated concentrations. Values show the mean from two technical replicates. (**D**) Bar graphs quantitating PAR and γH2AX immunofluorescence intensity in the cell lines indicated treated with PARGi for 72 hr at the concentrations indicated. Values show the mean ± SEM from three independent experiments. * $p < 0.05$, *** $p < 0.001$ and **** $p < 0.0001$.

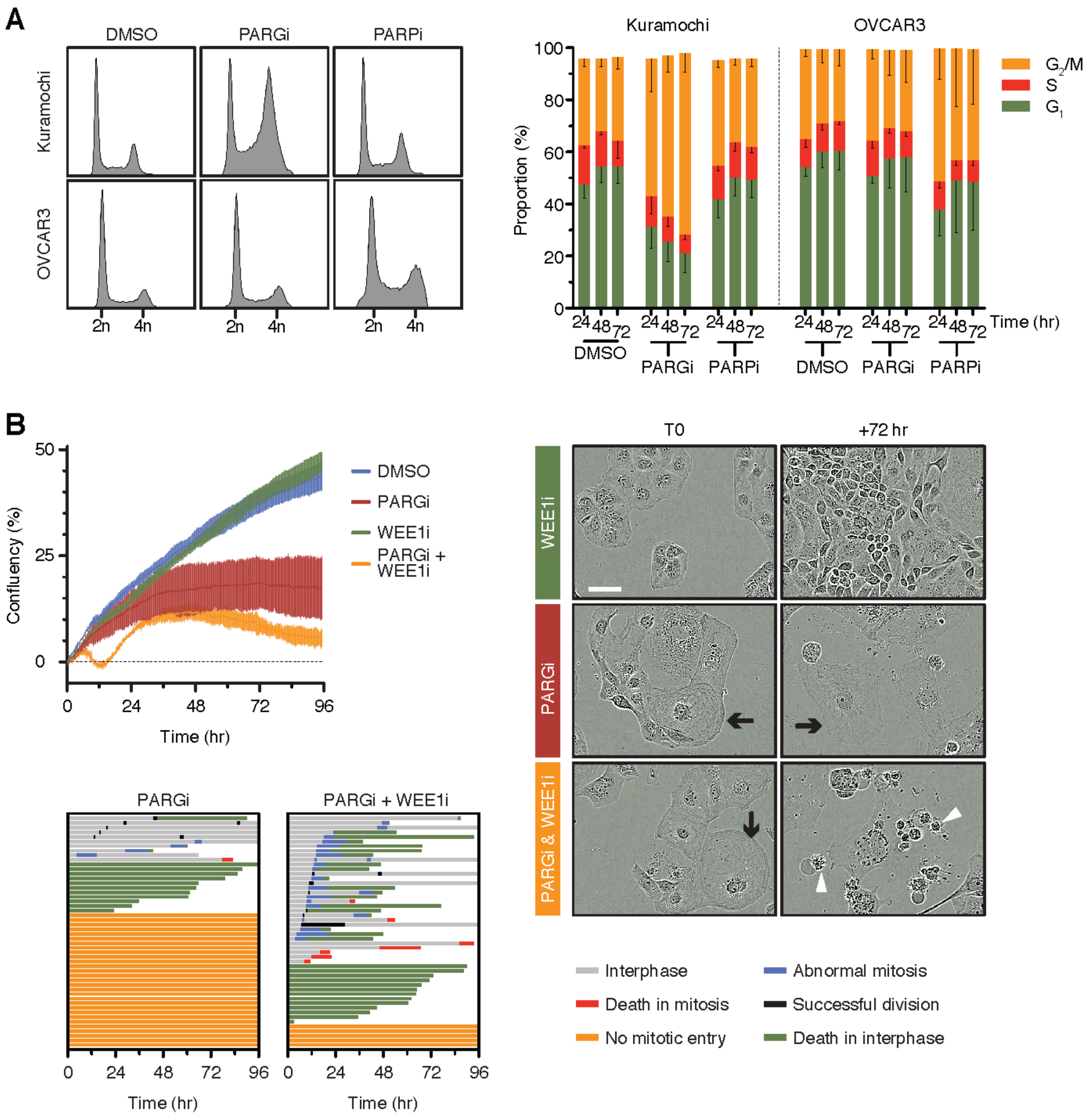

Figure S2, related to Figure 2. PARG inhibition blocks entry into mitosis. (A) DNA content histograms of Kuramochi and OVCAR3 cells exposed to PARGi or PARPi for 48 hr, plus bar graph quantitating the number of cells in G_1, S-phase and G_2/M; values derived from three independent experiments showing the mean ± SD. (**B**) Proliferation curves, cell fate profiles and phase contrast images derived from time-lapse imaging of Kuramochi cells treated with PARGi for 48 hr then exposed to the WEE1 inhibitor, AZD1775 (200 nM), hereafter WEE1i. Values on proliferation curves show the mean ± SD derived from three technical replicates. Note that the WEE1i induces the confluency of the PARGi-treated cells to dip at ~12 hr, corresponding to the cells being driven into mitosis. Confluency then increases as the cells return to interphase before decreasing again as cells undergo apoptosis. The cell fate profiles show that WEE1i overcomes the PARGi-induced mitotic block (orange) and increases apoptosis (red and green). The phase contrast images show PARGi-arrested cells at T_0, identified by "fried egg" appearance (arrows), and apoptotic bodies (arrow heads) following exposure to WEE1i; scale bar 50 µm.

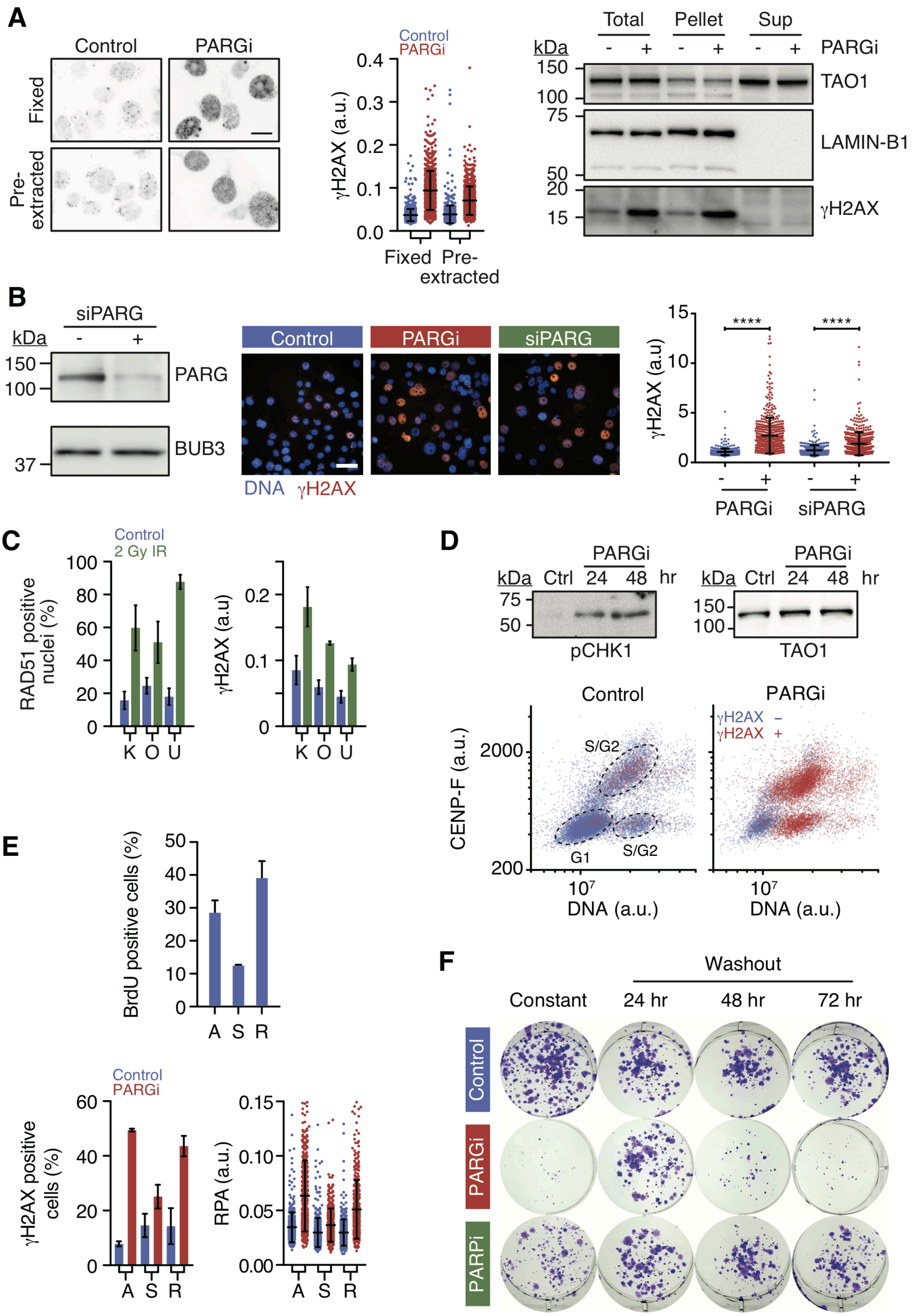

Figure S3, related to Figure 3. PARG inhibition induces replication catastrophe. (A) Immunofluorescence and immunoblot analysis of γH2AX in Kuramochi cells exposed to 1 μM PARGi for 48 hr. Scale bar 10 μm. Cells were pre-extracted or fractionated into pellets and supernatants to distinguish soluble and chromatin bound populations. Dot plot shows quantitation of γH2AX staining of 1000 cells per condition, showing mean ± SD of one independent experiment. Immunoblot analysis used LAMIN-B1 and TAO1 as loading controls. (**B**) Immunoblot and immunofluorescence analysis following inhibition of PARG by siRNA. Immunoblot shows repression of endogenous PARG following transfection of Kuramochi cells with siRNAs targeting PARG; BUB3 was used as a loading control. Immunofluorescence images showing pan-nuclear γH2AX in Kuramochi cells exposed to PARGi or following transfection of siRNAs targeting PARG. Dot plot shows quantitation of γH2AX staining in 2,500 cells showing means ± SD. **** $p < 0.0001$. Scale bar 50 μm. (**C**) Bar graphs quantitating RAD51 and γH2AX in Kuramochi (K), OVCAR3 (O) and U2OS (U) cells following exposure to 2 Gy of ionizing radiation. RAD51

positive cells are defined as having more than five foci, scoring at least 240 cells. γH2AX was quantified in at least 1000 cells. Values represent mean ± SEM from three independent experiments. (**D**) Immunoblot showing the increase of phospho-CHK1 in response to 24 and 48 hr PARGi treatment in Kuramochi cells; TAO1was used as a loading control. Immunofluorescence scatter plot of Kuramochi cells treated with 1 μM PARGi for 48 hr, quantitating CENP-F and DNA (DAPI), and cells positive (red) or negative (blue) for γH2AX. (**E**) Asynchronous Kuramochi cells (A) were partially synchronized in G_1 by treating with 2% DMSO for 48 hr (S), then triggered to enter S-phase by release from the DMSO block (R). Bar graph quantitates BrdU-positive cells as determined by immunofluorescence, scoring ≥ 260 cells per condition and showing mean ± SEM from two independent experiments. Populations were exposed to 1 μM PARGi for 48 hr then analyzed by immunofluorescence microscopy to quantitate γH2AX and RPA1 positive cells, with values representing mean ± SEM of at least 5000 cells per condition from three independent experiments and mean ± SD of 1000 cells representative of 3 independent experiments, respectively. (**F**) Colony formation assay with Kuramochi cells either constantly exposed to 1 μM inhibitors or exposed for 24, 48 and 72 hr then fixed after 21 day.

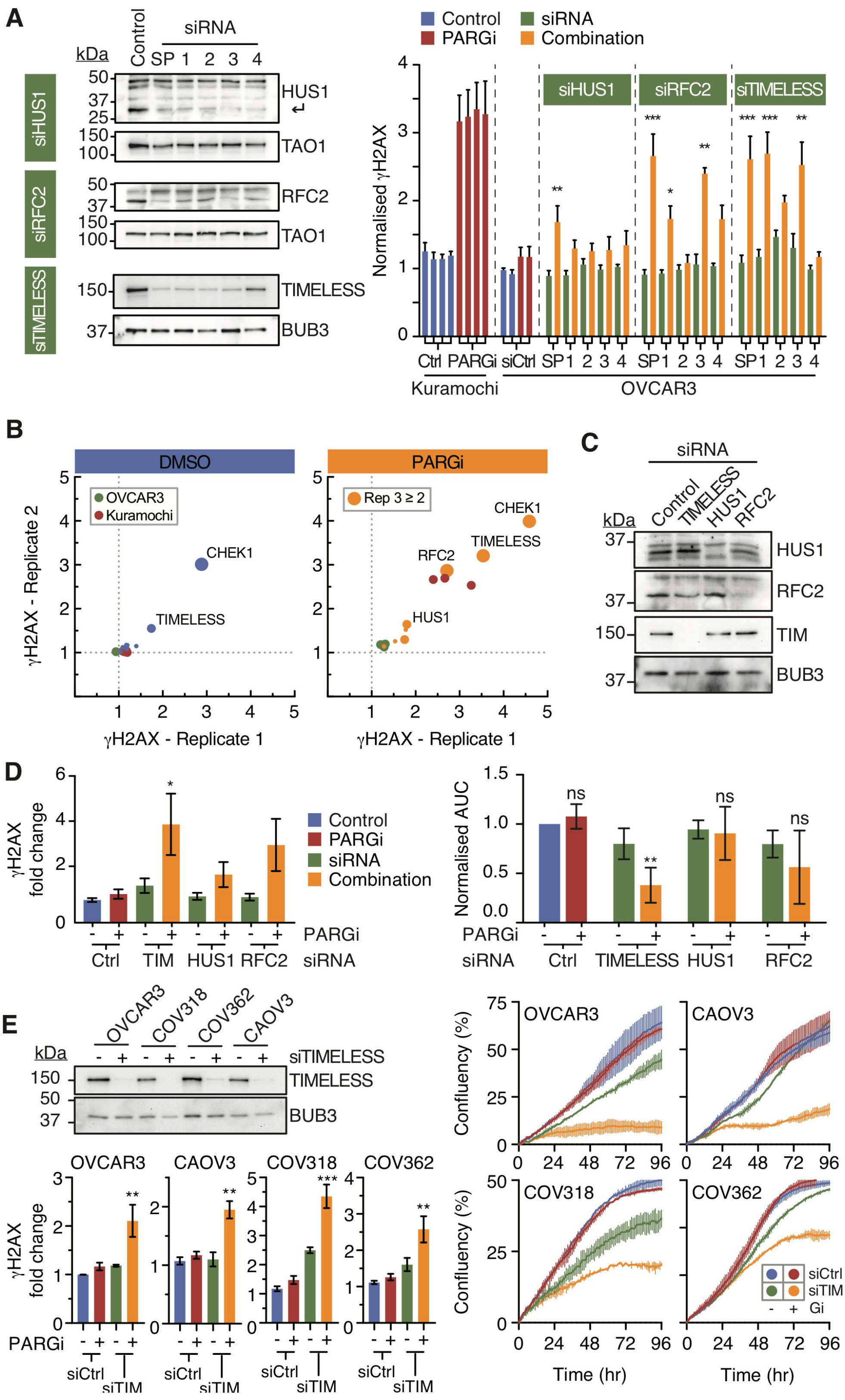

Figure S4, related to Figure 4. DNA replication factors are synthetic lethal with PARG inhibition. (**A**) Deconvolution of the siRNA SMARTpools. Immunoblots of OVCAR3 cells following transfection of the siRNA SMARTpools (SP) and the four individual, deconvolved siRNAs to identify those that inhibit HUS1, RFC2 and TIMELESS; TAO1 and BUB3 are used as loading controls. Bar graphs quantitating γH2AX staining in PARGi-treated cells following transfection of the SMARTpools and the individual siRNAs. Values show mean ± SEM derived from three independent experiments. * $p < 0.05$, ** $p < 0.01$, *** $p < 0.001$. (**B**) Secondary screen plotting γH2AX values for replicates 1 and 2, showing that siCHK1 induces γH2AX in the absence of PARGi. Values ≥ 2 in replicate 3 are denoted by a larger symbol. (**C**) Immunoblot, using BUB3 as a loading control, and (**D**) γH2AX quantitation and proliferation (normalized area under the curve) following RNAi-mediated inhibition of TIMELESS, HUS1 and RFC2 and exposure to PARGi for 48 hr. Values show the mean ± SEM from five independent experiments. ns - not significant, * $p < 0.05$, ** $p < 0.01$ (**E**) Analysis of RNAi-mediated inhibition of TIMELESS in the indicated cell lines; BUB3 used as a loading control. γH2AX values show the mean ± SEM from three independent experiments. Confluency-based proliferation curves show the mean ± SD from two technical replicates, representative of three independent experiments. ** $p < 0.01$, *** $p < 0.001$.

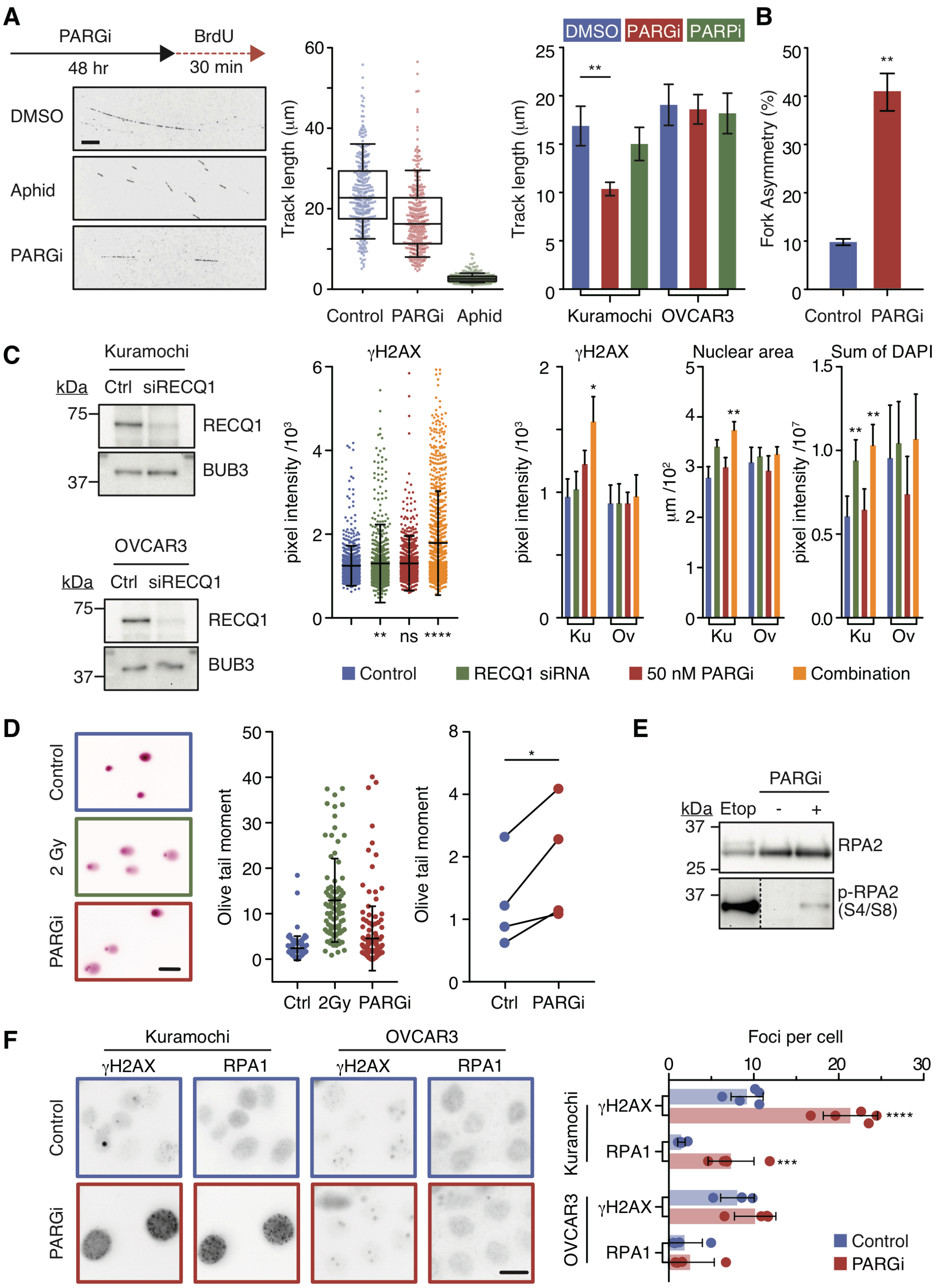

Figure S5, related to Figure 5. PARG inhibition induces replication fork asymmetry. (**A**) Experimental design, exemplar images and quantitation of at least 500 DNA fibers in control cells and following exposure to 0.58 µM aphidicolin or 1 µM PARGi. Scale bar 10 µm. Box-and-whiskers show median, interquartile and 10-90% ranges. Bar graph shows the mean track length ± SEM, derived from three independent experiments. ** $p < 0.01$. (**B**) Bar graph quantitating fork asymmetry in PARGi-treated Kuramochi cells, with values showing the mean ± SEM derived from three independent experiments. ** $p < 0.01$. (**C**) Effect of RNAi-mediated inhibition of RECQ1; immunoblots showing knockdown with BUB3 as a loading control, plus graphs quantitating γH2AX, nuclear area and sum of DAPI with and without 50 nM PARGi. Scatter plot shows effect on γH2AX in at least 1000 cells; bar graphs show the mean ± SEM of three independent experiments. * $p < 0.05$, ** $p < 0.01$, **** $p < 0.0001$ are all relative to the control (blue). (**D**) Comet assay analysis of Kuramochi cells exposed to 1 µM PARGi for 48 hr. Images show representative examples with 2 Gy ionizing radiation as a positive control; scale bar 100 µm. Scatter plot measures the olive tail moment from at least 80 cells showing the mean ± SD in one representative experiment. The before-after plot shows the mean values derived from four independent experiments. * $p < 0.05$. (**E**) Immunoblot of total RPA2 and

phospho-RPA2 (S4/S8) in Kuramochi cells following a 48 hr exposure to PARGi, or 25 μM etoposide for 4 hr as a positive control. The vertical line indicates deletion of an intervening lane. (**F**) Immunofluorescence images of cells exposed to 1 μM PARGi for 48 hr then stained to detect γH2AX and RPA1. Scale bar 20 μm. Quantitation of foci per cell shows the individual values and mean ± SEM derived from at least three independent experiments. *** $p < 0.001$, **** $p < 0.0001$, relative to the respective control.

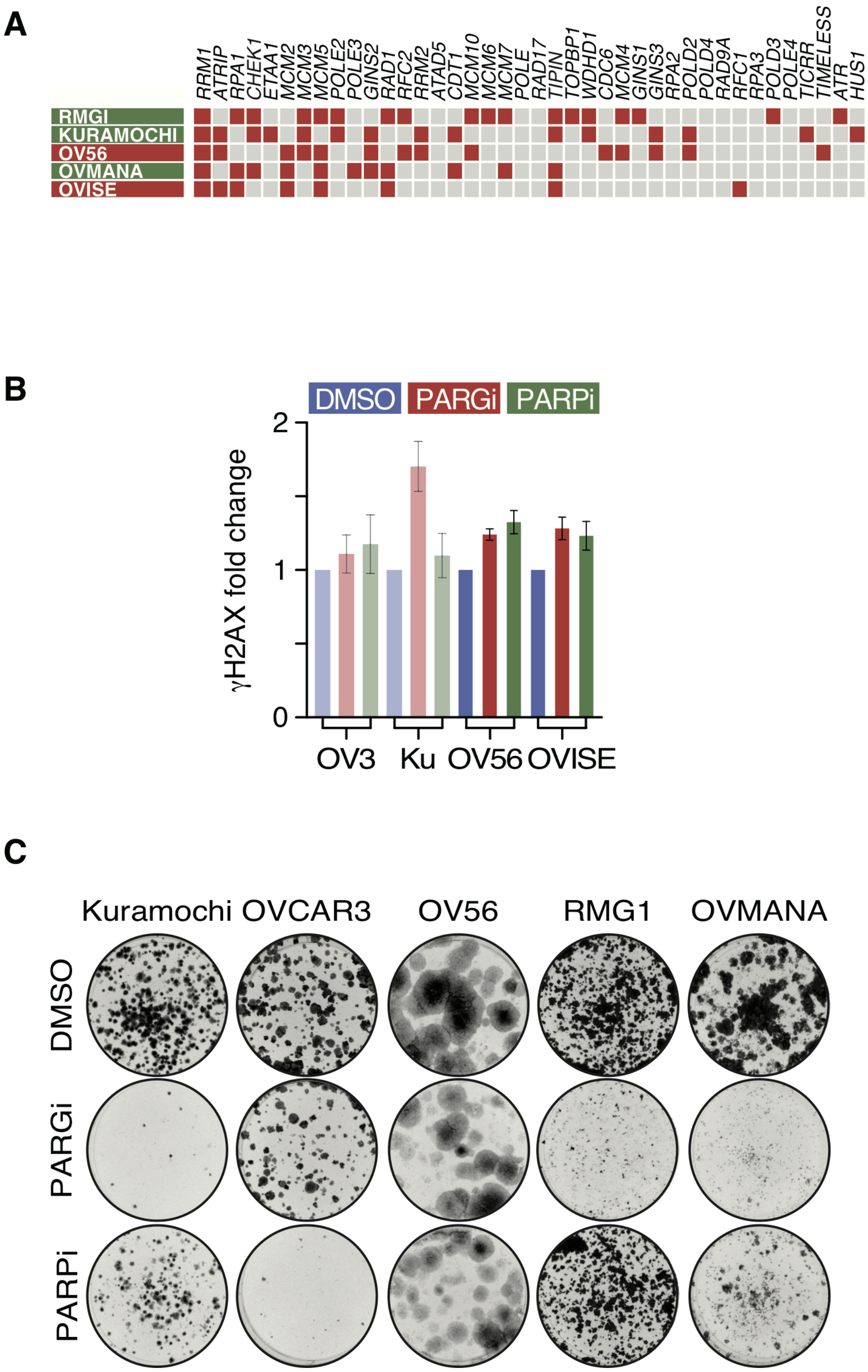

Figure S6, related to Figure 6. Interrogating DNA replication gene expression identifies additional PARG inhibitor sensitive lines. (**A**) DNA replication genes interrogated in ovarian cancer cell lines. (**B**) Bar graph quantitating γH2AX in response to 1 μM PARGi. Values show mean ± SEM, derived from three independent experiments. Note that values for OVCAR3 and Kuramochi cells are as shown in Figure 6C. (**C**) Colony formation in the continuous presence of 1 μM PARGi and 1 μM PARPi, representative of three independent experiments.

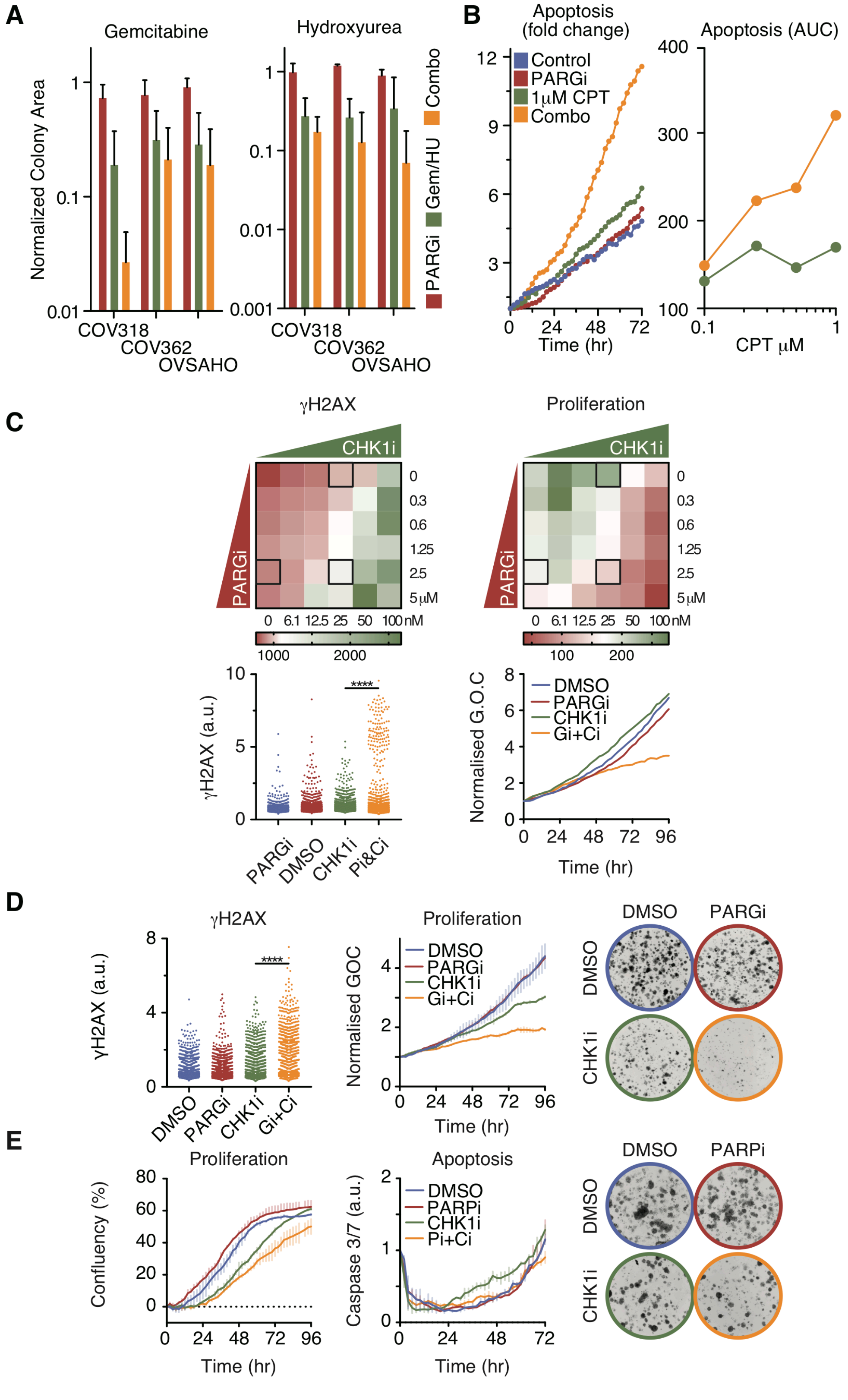

Figure S7, related to Figure 7. Replication stress sensitizes cells to PARG inhibition. (**A**) Quantitation of colony formation assays with bar graphs showing normalized colony area values derived from three independent experiments. Values show mean ± SD. (**B**) Fold change in apoptosis, measured by propidium iodide fluorescence in COV318 cells exposed to 1 μM PARGi and 1 μM camptothecin (CPT) and area under the curve measurements at 0.1, 0.25, 0.5 and 1 μM CPT. (**C**) Heatmaps measuring γH2AX and proliferation (area under curve) following a 96 hr exposure to combinations of PARGi and CHK1i in OVCAR3 cells. Dot plot quantitating at least 1500 cells and proliferation curves (average of two technical replicates) showing measurements at the concentrations highlighted by black boxes in heatmaps. **** $p < 0.0001$. (**D**) γH2AX quantitation of 1000 cells, proliferation curves, and colony formation assay of COV318 treated with 1 μM PARGi and 25 nM CHK1i. Proliferation values show mean ± SD from two technical replicates. Each panel representative of three independent experiments. **** $p < 0.0001$. (**E**) Proliferation curves, apoptosis and colony formation assay for OV56 exposed to 75 nM CHK1i and 1 μM PARPi. Proliferation and apoptosis values show mean ± SD from two technical replicates, and each panel is representative of three independent experiments. Note that the OV56 DMSO and CHK1i images are shown in Figure 7.

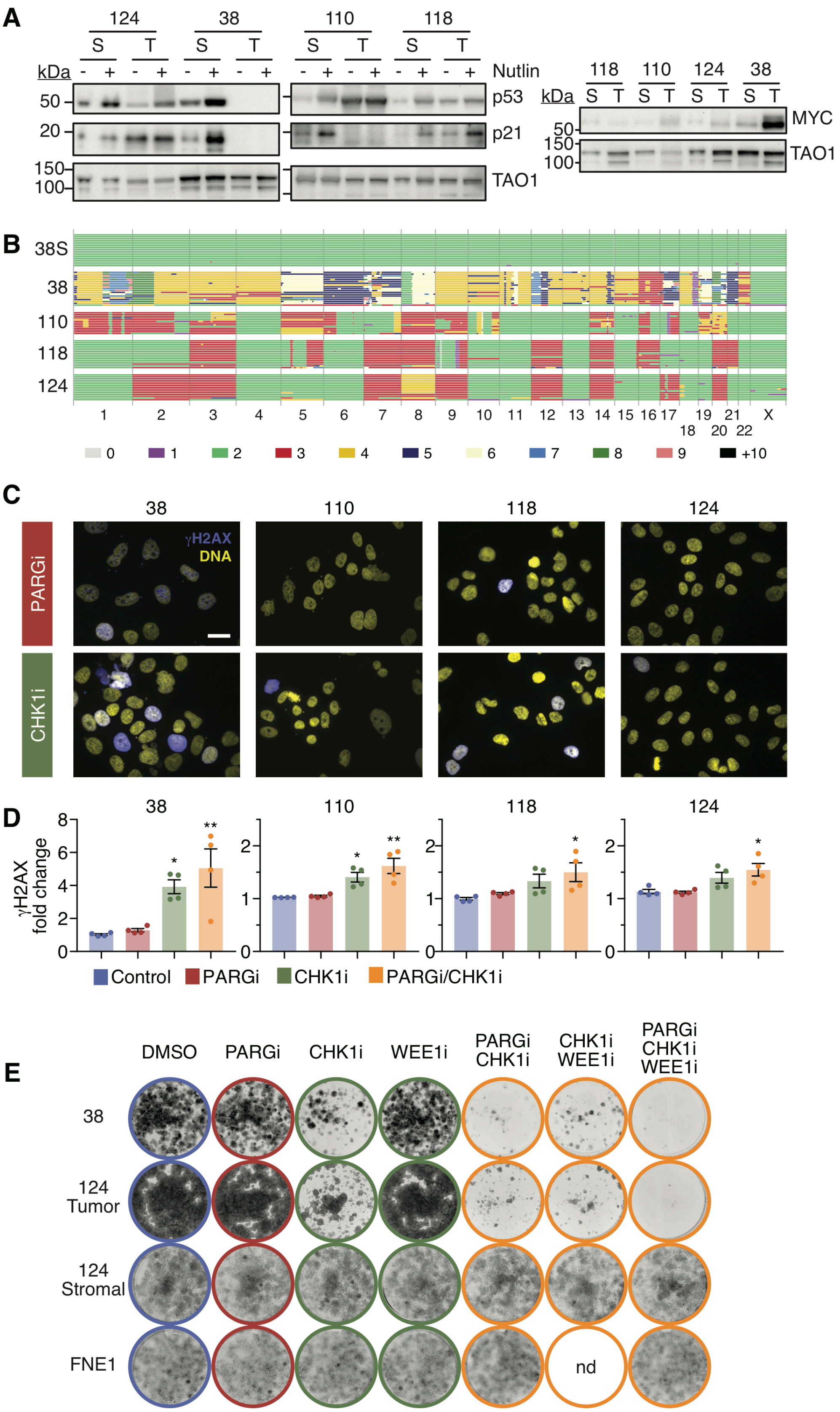

Figure S8, related to Figure 8. Validation of patient-derived ovarian cancer models. (**A**) Immunoblots analyzing p53, p21 and MYC in paired stromal (S) and tumor (T) *ex vivo* cell cultures. p53 and p21 are analyzed in the presence and absence of the MDM2 inhibitor, Nutlin-3. TAO1 is used as a loading control. (**B**) Genome-wide chromosome copy number profiles determined by single-cell whole genome sequencing showing aneuploidies and rearranged chromosomes in the tumor cells. Each row represents a single cell, with chromosomes plotted as columns and colors depicting copy number state. (**C**) Additional controls for Figure 8C, showing γH2AX immunofluorescence images of patient biopsy-derived cells exposed to 1 µM PARGi and CHKi (38, 200 nM; 110, 100 nM; 118, 50 nM; 124, 50 nM). Scale bar 30 µm. (**D**) Bar graphs quantitating γH2AX fold change, showing the individual values, the mean ± SEM derived from four technical replicates. * $p < 0.05$, ** $p < 0.01$ (**E**) Colony formation assays of cells treated with the drug combinations (1 µM PARGi, 50 nM WEE1i and 200 nM (38) or 75 nM (124 tumor, 124 stromal and FNE1) CHK1i for 96 hr then fixed after 12 day.

Table S1, related to Figure 8. Clinical data associated with ovarian cancer models.

OCM	Age at dx*	Anatomical site	Histology†	Grade	FIGO stage	g*BRCA1/2*	p53 IHC staining	Other positive IHC staining	CTx	Plt sensitivity	PFI
110	57	OV	Serous	HG	3C	WT	Diffuse, strong	CK7, PAX8, WT1	C/T (6Cy)	Plt-Res	2.5
118	41	PP	Serous	LG^	3C	WT	Heterogeneous†	CK7, PAX8, WT1, ER	C/T (3Cy)	Plt-Ref	NA
124	56	OV	Serous	LG^	3C	WT	Heterogeneous†	CK7, PAX8, WT1, ER	C/T (4Cy)	Plt-Ref	NA
38	81	OV/PP	Serous	HG	3C	Not tested	Diffuse, strong	CK7, PAX8, WT1	C/T (6Cy)	Plt-Res	4.7

Key: OV=ovarian; PP=primary peritoneal; OV/PP ovarian or primary peritoneal; HG= high-grade (poorly differentiated; grade 3); LG= low-grade (well differentiated; grade 1); FIGO= International Federation of Gynecology and Obstetrics; WT= wild type; NA= not applicable; IHC= immunohistochemistry; C/T= carboplatin plus paclitaxel; Plt= platinum; Plt-Res= platinum-resistant (PFI<6 month); Plt-Ref= platinum refractory (progressive disease whilst receiving first-line platinum-based chemotherapy); PFI= platinum-free interval; Cy= cycles; CTx= first-line chemotherapy; *Age in year; †Adenocarcinoma; † equivalent to "wild type" staining; g*BRCA1/2* = germline *BRCA1* or *BRCA2* pathogenic/likely pathogenic variant; ^Both samples had features of nuclear atypia.
